# Androgen Deprivation-Induced Senescence Promotes Outgrowth of Androgen-Refractory Prostate Cancer Cells

**DOI:** 10.1371/journal.pone.0068003

**Published:** 2013-06-28

**Authors:** Dominick G. A. Burton, Maria G. Giribaldi, Anisleidys Munoz, Katherine Halvorsen, Asmita Patel, Merce Jorda, Carlos Perez-Stable, Priyamvada Rai

**Affiliations:** 1 Department of Medicine, University of Miami Miller School of Medicine, Miami, Florida, United States of America; 2 Biology Department, University of Miami, Coral Gables, Florida, United States of America; 3 Department of Pathology, University of Miami Miller School of Medicine, Miami, Florida, United States of America; 4 Bruce W. Carter Veterans Affairs Medical Center, Miami, Florida, United States of America; 5 Sylvester Comprehensive Cancer Center, University of Miami Miller School of Medicine, Florida, United States of America; Florida International University, United States of America

## Abstract

Androgen deprivation (AD) is an effective method for initially suppressing prostate cancer (PC) progression. However, androgen-refractory PC cells inevitably emerge from the androgen-responsive tumor, leading to incurable disease. Recent studies have shown AD induces cellular senescence, a phenomenon that is cell-autonomously tumor-suppressive but which confers tumor-promoting adaptations that can facilitate the advent of senescence-resistant malignant cell populations. Because androgen-refractory PC cells emerge clonally from the originally androgen-responsive tumor, we sought to investigate whether AD-induced senescence (ADIS) affects acquisition of androgen-refractory behavior in androgen-responsive LNCaP and LAPC4 prostate cancer cells. We find that repeated exposure of these androgen-responsive cells to senescence-inducing stimuli via cyclic AD leads to the rapid emergence of ADIS-resistant, androgen-refractory cells from the bulk senescent cell population. Our results show that the ADIS phenotype is associated with tumor-promoting traits, notably chemoresistance and enhanced pro-survival mechanisms such as inhibition of p53-mediated cell death, which encourage persistence of the senescent cells. We further find that pharmacologic enforcement of p53/Bax activation via Nutlin-3 prior to establishment of ADIS is required to overcome the associated pro-survival response and preferentially trigger pervasive cell death instead of senescence during AD. Thus our study demonstrates that ADIS promotes outgrowth of androgen-refractory PC cells and is consequently a suboptimal tumor-suppressor response to AD.

## Introduction

Prostate cancer (PC) is one of the most common malignancies in men and a leading cause of cancer-related deaths. For advanced disease, androgen deprivation therapy is the main treatment regimen due to the critical dependence of prostatic tissue on androgens for proliferation and survival [1]. However the eventual emergence of androgen-refractory tumors, which no longer respond favorably to androgen withdrawal, reduces patient life expectancy to less than two years because these tumors are poorly responsive to additional treatments [2], [3]. The molecular mechanisms that give rise to these androgen-refractory tumors are not well understood. Substantial research has focused on androgen receptor (AR) signaling and implicates AR-related aberrations including gene amplification, ligand-independent or promiscuous ligand-based activation and altered co-regulator expression [2], [4]–[7]. Non-AR pathways thought to be involved in androgen-refractory proliferation include bypass of AR-based proliferation control via oncogene activation or tumor suppressor downregulation, altered chromatin regulation of gene transcription, disruption of cell cycle control machinery and elevated expression of steroidogenic enzymes [8]–[14].

However, many studies investigating these mechanisms examine them in the context of advanced prostate cancer models and do not address the earliest changes required for the evasion of AD-induced tumor suppression. This is an important issue because, despite its high initial tumor-inhibitory effect [15], AD does not kill all androgen-responsive prostate cancer cells but rather induces a proliferative arrest in a significant subpopulation [16]. Considering that androgen-refractory cell populations are characterized by the acquisition or enhancement of stress-protective pro-tumorigenic pathways [8], [10], [17], the properties of these proliferation-arrested cells are likely to be important in understanding how androgen-refractory subpopulations arise. Indeed it is plausible that a subset of these arrested cells exit proliferative stasis and become non-responsive to AD. In support of this idea, it has been reported that prostate tumors consist of heterogeneous cell mixtures in terms of androgen response [18], and that androgen-refractory cells arise as a selected variant population from the parental androgen-responsive cells [18], [19]. Furthermore, consistent with the clinical phenotype, prolonged (>6 months) *in vitro* androgen-ablated culture of androgen-responsive prostate cancer cell lines leads to the eventual outgrowth of androgen withdrawal–resistant clones from the initially growth-arrested population [8], [20]. Therefore, elucidating the molecular mechanisms that underlie the AD-induced growth arrest is critical for defining the etiology of androgen-refractory PC.

The molecular characteristics of the AD-induced growth arrest include a G1/S block, reduced cyclin-dependent kinase activity, hypophosphorylated Rb, and abrogation of the arrested phenotype via introduction of the oncoprotein, SV40 Large T Antigen [21]. These traits are consistent with the AD-induced arrest being a form of cellular senescence [22], and two recent studies have reported that androgen-deprived cells develop molecular markers consistent with senescence [23], [24]. Models of oncogene-driven tumorigenesis have demonstrated that oncogene-induced senescence leads to selective pressures that promote outgrowth of senescence-resistant aggressive tumor cell subpopulations [25]–[27]. However, this tumor-promoting aspect of senescence has not, to our knowledge, been previously explored for hormone withdrawal-associated senescence. We therefore designed our study to determine whether a similar paradigm, namely evasion of AD-induced senescence (ADIS), operates in the generation of androgen-refractory prostate cancer cells from the parental androgen-responsive population. Accordingly, in this study, we utilized the LNCaP and LAPC4 PC cell lines, known to possess the major salient features of androgen-responsive prostatic tumor cells including robust androgen receptor and prostate specific antigen expression, enhanced proliferation in response to androgens and proliferative cessation upon androgen withdrawal, and inability to form tumors in castrated mice. We cultured these cells in charcoal-stripped serum (CSS)-containing media to recapitulate AD in culture. Our objective was to characterize the molecular pathways associated with ADIS and to determine whether we could isolate senescence-resistant variants able to proliferate under AD.

Our results demonstrate that ADIS leads to acquisition of tumor-promoting traits such as enhanced pro-survival mechanisms and chemoresistance. Significantly, sustained presence of senescence-inducing stimuli via cyclic AD facilitates expansion of ADIS-resistant androgen-refractory LNCaP and LAPC4 variants within a period of weeks. These variants are partially imprinted with senescence-associated pro-survival features despite being ADIS-resistant. Thus, our findings collectively support a role for the senescent phenotype in engendering changes that promote emergence of androgen-refractory tumor cells.

## Materials and Methods

### Ethics Statement

All research involving human subjects has been approved by the University of Miami Institutional Review Board. The IRB approved waiver of consent for this protocol.

### Cell Culture

LNCaP and LAPC4 cells (ATCC) were cultured in RPMI-1640 medium (Gibco, Invitrogen) supplemented with 5% fetal bovine serum (FBS; Gibco, Invitrogen) or 5% charcoal-stripped fetal bovine serum (CSS; Gibco, Invitrogen) and 100 units/ml penicillin/streptomycin (Gibco, Invitrogen) at 37°C in 21% oxygen/5% CO_2_. For quiescent cultures, cells were cultured as above but in the absence of any serum.

### Switchback (SB) Method to Generate Androgen-refractory ADIS-resistant LNCaP Variants

LNCaP cells were seeded into either 10 cm dishes (3×10^5^ cells) or 15 cm dishes (1×10^6^ cells) in RPMI 5% FBS for 36–48 hours before being switched to RPMI 5% CSS. Media was replaced every 3–4 days for 14 days, to induce ADIS, subsequent to which the cultures were restored to RPMI 5% FBS media. Upon outgrowth of visible cell colonies, cells were trypsinized and expanded for one or two passages before the procedure was repeated.

### Creation and Stable Transduction of shRNA and Fluorescent Protein-expressing Constructs

The retroviral pWZL-blast GFP construct was a gift from Dr. Robert Weinberg’s laboratory. The shRNA vectors were generated as described previously [28], [29]. Validated or highly scored candidate sequences from the TRC Broad Consortium shRNA library (http://www.broadinstitute.org/genome_bio/trc/publicSearchForHairpinsForm.php) were selected and subcloned into the lentiviral pLKO.1 vector with a puromycin resistance cassette. Oligonucleotides were purchased from Integrated DNA Technologies, Inc. Constructs were sequenced to ensure presence of the insert. The control shRNA was targeted against GFP: 5′-GCAAGCTGACCCTGAAGTTCA-3′. The following target sequences were used:

shp53: 5′GACTCCAGTGGTAATCTACTT 3′,

shp16: 5′GCATGGAGCCTTCGGCTGACT 3′

The shAR construct [30] was a gift from Dr. Kerry Burnstein at the University of Miami and was targeted against the following sequence: 5′ GAAAGCACTGCTACTCTTCAGCATTATTC 3′.

Lentiviral or retroviral production was carried out in HEK 293T cells and infection of target cells were performed as described previously [29]. Transduced cells were selected in 2 µg/ml puromycin-containing or 10 µg/ml blastocidin-containing media (for SB5 GFP cells) for a minimum period of 5–7 days (corresponding to the time taken for untransduced cells to die completely in selection media). For shRNAs, protein knockdown was verified via Western blotting. GFP expression in the SB5 cells was verified by epifluorescent imaging and flow cytometric analysis.

### Western Blotting and Antibodies Used

Cells were harvested by mechanical scraping on ice and were lysed in a sodium fluoride (NAF) buffer containing sodium vanadate, PMSF, DTT and proteinase inhibitor. Protein concentrations were measured using the Bradford reagent (Biorad). 30–50 µg of total protein was run on a 4–12% Bis-Tris pre-cast NuPage gel (Invitrogen) on the Novex precast gel system and subsequently transferred onto a section of PVDF membrane (Immobilon, EMD Millipore) at 30 V at 4°C. Blots were stained in Ponceau reagent to determine even loading and transfer, washed in 0.1% TBST and then probed with antibodies against the following proteins: AR (sc-816, Santa Cruz Biotech), p53 (sc-126, Santa Cruz Biotech), p21 (sc-817, Santa Cruz Biotech) p16^INK4^ (554079, BD Biosciences), cyclin A (sc-751, Santa Cruz Biotech), GAPDH (AB9485, Abcam) Bcl-2 (sc-492, Santa Cruz Biotech), Bax (sc-493, Santa Cruz Biotech) Mcl-1 (sc-819, Santa Cruz Biotech), Bak (06-536, Millipore), phospho-Akt (4060, Cell Signaling) total Akt (9272, Cell Signaling) cleaved-PARP (9541, Cell Signaling), survivin (71G4B7, Cell Signaling), TAp63 (618902, BioLegend), p27 (sc-528, Santa Cruz Biotech), deltaNp63 (619002, BioLegend). Following incubation with the appropriate secondary horseradish peroxidase-conjugated antibodies (Amersham), blots were developed using the ECL Plus (GE Healthcare) developing solution. Following immunoblotting, membranes were stained with Coomassie blue dye (Sigma) to normalize for total protein loading.

### Cell Cycle Analysis

Approximately 1×10^6^ cells were harvested via trypsinization. Cells were washed once in cold serum-containing medium, twice in cold PBS and then resuspended in 200 ul of PBS +2 mM EDTA. Approximately 600 µl of cold 70% ethanol was added dropwise to cells while vortexing them at medium speed. Cells were kept at 4°C overnight for fixation. Prior to analysis, cells were spun down at 4°C at 1000 rpm for 5 minutes and the ethanol carefully aspirated. The resulting cell pellets were resuspended in 0.5 ml of PBS +2 mM EDTA and 10 µl heat-inactivated RNase A (10 mg/ml, Sigma). Thereafter, 25 µl propidium iodide (PI) (1 mg/ml, Sigma) was added to the cell suspension. Cells were incubated at 37°C in a water bath for 30 minutes and immediately assayed on an Accuri C6 flow cytometer (BD) using the PI excitation laser (FL2). The percentage of cells in each phase of the cell cycle was determined on the CFlow Plus cytometer software, CFlow Analysis 202.1, by measuring area under the relevant segment of the profile (as shown). Profiles were relabeled in MS Excel for clarity.

### Cell Proliferation Measurements

**Figure 1:**
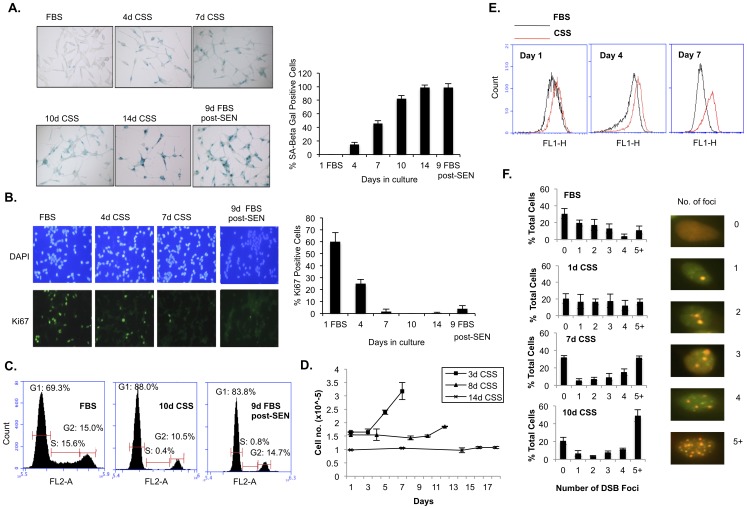
Androgen deprivation induces senescence markers in LNCaP cells. (**A**) Senescence associated beta-galactosidase (SA-beta-gal) staining under indicated conditions and timepoints. FBS, fetal bovine serum indicating androgen-replete culture; CSS, charcoal-stripped serum indicating androgen- deprived culture. 9D FBS post-SEN denote LNCaP cells that were subjected to 14 days CSS culture and then switched to FBS media for 9 days. Quantitation of positively stained cells is presented to the right of the representative images. (**B**) Ki67 pan-proliferation marker staining is shown at indicated conditions and time points. DAPI staining is used to mark cell nuclei for purposes of counting Ki67-positive cells. Percentage of stained cells is graphed on the right. (**C**) Propidium iodide cell cycle analysis, at indicated timepoints and culture conditions. Note the S-phase fraction drops from 15.6% in FBS to 0.4% at 10d CSS and to 0.8% after restoration to FBS media for 9 days (9D FBS post-SEN). (**D**) Proliferation curve for LNCaP cells cultured in CSS for 3, 8 or 14 days before being switched back to FBS media. (**E**) Measurement of total ROS levels via flow cytometry in LNCaP cells at day 1, day 4 and day 7 of indicated culture conditions. Note the right-shifted peak in red corresponding to increasing ROS levels in the CSS-cultured LNCaP cells relative to FBS-cultured cells. (**F**) DNA double-strand break (DSB) foci detected via H2AX/53BP1 co-staining (green = H2AX, red = 53BP1) for LNCaP cells cultured under the indicated conditions. Representative images for cells with the different numbers of counted foci are shown. Note that the percentage of cells with 5+ foci increases with the duration of CSS culture.

To determine cell proliferation rates of LNCaP cells in both FBS and CSS containing media, 1×10^5^ were seeded in 6 cm plates and cell counts, using a hemocytometer, were carried out every 24 hours, in triplicate over a six-day period using a hemocytometer. Fresh media was added every 2–3 days. For rescue experiments in Fig. 1D, cells were seeded in FBS media for 24 hrs before switching to CSS media. Cells were switched back to FBS media at the indicated time points.

### Cell Co-culture Experiments and Analysis

For the co-culture experiments, 1×10^5^ LNCaP-SB5-GFP cells (generated from SB0 LNCaP cells stably transduced with pWZL-blast GFP) were plated in quadruplicate in a 10 cm dish, either alone or co-mixed with 1×10^5^ unlabeled LNCaP SB0, SB5 or LNAI cells. Cells were initially plated in 5% FBS complete media (Day 0) and on Day 1, the co-cultures were switched to 5% CSS media and maintained for 7 days, with a CSS media change every three days. Representative cultures were analyzed in duplicate by flow cytometry on an Accuri C6 cytometer on Day 1 to ensure relatively equivalent numbers of green and non-green cells were surviving in culture. Using the SB5-GFP cells plated alone, gating was accomplished on the FITC channel to separate the GFP cells from non-GFP cells. Cells were further gated to exclude low FSC/SSC particles to ensure only live cell populations were being analyzed. On day 7, flow cytometry was used to determine the relative percentages of GFP-positive versus unlabeled cell populations as well as the total number of GFP-positive (SB5) cells. Histograms and dot plots were plotted using the BD Accuri C6 Analysis software for Mac OSX. All measurements were carried out in duplicate in a minimum of two independent experiments.

### Senescence-associated Beta-galactosidase Activity (SA-beta-gal)

SA-beta-gal staining was carried out as described elsewhere [31]. Briefly, cells were washed in PBS, fixed in 0.2% glutaraldehyde for 5 min at room temperature, washed once in PBS and incubated overnight in freshly-made staining solution (1 mg/ml 5-bromo-4-chloro-3-indolyl-β-galactoside (Sigma), 150 mM NaCl, 2 mM MgCl_2_, 5 mM K_3_Fe (CN)_6_, 5 mM K_4_Fe (CN)_6_, 40 mM NaPi, pH 6.0). The following day, cells were incubated for a further hour at 37°C to intensify staining and then washed and stored in PBS at 4°C. To quantify positive staining, >100 cells were counted for each sample in several fields of view in order to obtain a standard deviation. Experiments were carried out at least in duplicate, in two independent experiments.

### Ki67 Staining

For detection of Ki67 staining, cells were plated at 2×10^4^ cells per chamber and left for 36–48 hours before either switching to CSS, bicalutamide treatment or fixation for FBS only samples. Cells were fixed in 4% paraformaldehyde (16% stock, Electron microscopy sciences) for 10 minutes at room temperature, followed by permeabilization in 0.1% PBST (10 min at RT), and blocked for 1 hour in 0.1% TritonX, 15% FBS, in PBS at RT. The Ki67 antibody (Santa Cruz) was diluted 1∶50 in blocking buffer and incubated at 4°C overnight. Cells were washed 5X, 10 minutes each time at RT in blocking solution on a shaker. The appropriate fluorophore-conjugated secondary antibody (goat anti-mouse-FITC, Santa Cruz) was added in a 1∶100 dilution in blocking buffer and incubated for 1 hour at RT in the dark. Cells were washed 5X in 0.1 TritonX, 10 minutes each time on shaker at RT in the dark. DAPI mounting media (Vectashield) was added before mounting coverslip. Immunofluorescent images were acquired on a Nikon camera attached to an upright Zeiss microscope.

### H2AX/53BP1 Staining

H2AX/53BP1 staining was carried out as described elsewhere [28]. Briefly, 20,000 cells were seeded per chamber (BD Falcon, 4-chamber culture slides) in RPMI 5% FBS and switched to RPMI 5% CSS 24 hours later. H2AX/53BP1 staining was carried out at the indicated time points. Cells were photographed under identical acquisition conditions on a Leica DMI 6000 B inverted digital microscope with an attached DFC350 FX camera and FW4000 I fluorescence software. Only co-localized H2AX/53BP1 foci were counted as positive and a minimum of 100 cells were scored over multiple fields.

### Drug Treatment of LNCaP Cells

LNCaP cells were cultured in appropriate media (5% FBS or CSS) containing docetaxel (1 nM), flavopiridol (0.5 µM), or bortezomib (1 µM) for 72 hours, and floating and trypsinized attached cells were pooled and analyzed by Trypan blue staining. LNCaP-SB0 and LNCaP-SB5 cells were cultured in media (RPMI +5% FBS) containing docetaxel (1 nM) for 72 hours and analyzed by Trypan blue staining. At least 3 independent experiments were done in duplicate and statistical differences between drug-treated cells were determined by two-tailed Student’s *t*-test with p<0.05 considered significant.

### Reverse-transcriptase PCR

Total RNA was isolated from LNCaP cells cultured in FBS and CSS media for 14 days, using the RNAqueous-4PCR Kit (Ambion). A total of 1 µg purified RNA was reverse-transcribed using a random primer, included in the kit, to obtain cDNA via the High Capacity cDNA Reverse Transcription Kit (Applied Biosystems). The cDNA was used as a template for PCR amplification using AmpliTaq Gold® 360 Master Mix (Applied Biosystems) and the primers were as follows.

GAPDH forward primer:

5′ GACCCCTTCATTGACCTCAAC 3′

GAPDH reverse primer:

5′ CTTCTCCATGGTGGTGAAGA 3′

IL8 forward primer:

5′ TTTTGCCAAGGAGTGCTAAAGA 3′

IL8 reverse primer:

5′ AACCCTCTGCACCCAGTTTTC 3′

The reaction products were run on 1.5% agarose gel with ethidium bromide. GAPDH was used as an internal normalization control.

### Measurement of Cell Death

Treated and control cells were harvested, resuspended in PBS, and diluted 1∶1 in 0.4% Trypan blue (Invitrogen). Dead (blue) and live non-stained cells immediately counted using a hemacytometer. The percentage of dead cells was determined from at least three independent experiments done in duplicate.

### Measurement of Cellular ROS Levels

The assay was carried out as described previously [28]. Briefly, the indicated cells at equivalent confluency were collected through trypsination, washed in ice-cold 1X Hank’s buffered saline solution (HBSS) and incubated with freshly prepared 5- (and-6)-chloromethyl-2′,7′-dichlorofluorescein diacetate (CM-DCF-DA; Molecular Probes/Invitrogen, C6827) for 20 min at 37°C. The cells were then washed and resuspended in 1X HBSS before detection of FITC signal through fluorescence-activated cell sorting (FACS). Flow cytometric analysis was conducted on an Accuri C6 cytometer (BD Biosciences). The x-axis represents FITC channel (FL1) fluorescence intensity in log-scale and the y-axis represents the number of cells. Experiments were carried out at least in duplicate, with representative profiles being shown.

### Crystal Violet Staining

Approximately 3×10^5^ cells were seeded into 10 cm dishes containing RPMI plus 5% FBS for 36–48 hrs before switching to RPMI plus 5% CSS. Media was refreshed every 3–4 days. For crystal violet staining, media was aspirated and cells washed once in 1X PBS, 1% crystal violet solution was added to each dish and left to stain for 2 minutes at room temperature. The stain was removed and each dish rinsed twice in deionized water. Dishes were then submerged in water for 20–30 minutes to leach excess stain and reduce background, and left to air-dry overnight before counting colonies or taking pictures. Experiments were carried out in duplicate.

### Statistical Analysis

Statistical significance of results was determined by calculating standard deviation from the mean and by a two-tailed Student’s t-test, with p<0.05 considered significant.

## Results and Conclusions

### Characteristics of ADIS in LNCaP and LAPC4 Cells

In order to confirm whether the observed proliferative arrest upon AD is due to the induction of cellular senescence, we utilized the well-characterized androgen-sensitive cell line LNCaP as it possesses functional versions of both major senescence-mediating pathways, p53 and p16^INK4a^ [32], [33]. Furthermore, congruent with clinical models of prostate cancer, LNCaP cells show spontaneous outgrowths of androgen-refractory variants following long-term culture in androgen-depleted media [20], [34]. Accordingly, we cultured LNCaP cells in CSS-containing media (hereafter referred to as CSS culture) to mimic AD and assessed emergence of cellular senescence markers.

We found that in addition to the expected proliferative arrest (Fig. S1A) and acquisition of a neuroendocrine morphology [35] (Fig. 1A), CSS-cultured LNCaP cells also developed other distinct markers of cellular senescence (Fig. 1) [36]. These included increased senescence-associated beta-galactosidase (SA-beta-gal) activity (Fig. 1A), decreased staining for the pan-proliferation marker, Ki67 (Fig. 1B) and a G1/S cell cycle arrest (Fig. 1C). After 7 days in CSS culture, >50% cells exhibited these characteristics and by 10 days in CSS, that fraction rose to >80% of the bulk population (Fig. 1A–B). Treatment with the anti-androgen, bicalutamide, also induced proliferative cessation and the appearance of a senescent phenotype as determined by SA-beta-gal staining and a G1/S arrest (Fig. S1). By contrast, minimal cell death was observed upon CSS or bicalutamide treatment as judged by a visual lack of rounded, unattached cells and cleaved PARP expression (data not shown). Significantly when the cells were restored to androgen-replete fetal bovine serum (FBS) media following 14 days of CSS culture, the bulk population retained senescent markers (shown in the case of restoration to replete FBS culture for 9 days, denoted as 9D FBS post-SEN), indicating that the proliferation arrest is permanent rather than transient in nature (Fig. 1A–C).

To further distinguish the CSS culture-induced proliferation arrest from the temporary phenomenon of cell quiescence, we cultured LNCaP cells in serum-free media, known to induce quiescence [37], for 7 days in parallel with cells cultured in CSS media. Thereafter we switched both sets of cells back to FBS media for 4 days. Whereas the LNCaP cells subjected to serum-free culture resumed proliferation upon restoration to full media, corresponding to an increase in the proliferation marker, cyclin A, the CSS-cultured cells did not do so (Fig. S2). As the LNCaP cells undergo no further population doublings once placed under androgen-deprived culture (Fig. S1A) but take up to 4 days to show significant acquisition of senescence markers (Fig. 1A, B), we wanted to determine if this proliferation arrest was reversible prior to 4 days of androgen depletion. To do so, LNCaP cells were switched back to androgen-replete media at 3, 8 or 14 days post-CSS culture (Fig. 1D). We found that cells switched back to FBS media at day 3 were readily able to resume proliferation. In comparison, cells switched to replete media at day 8 did not resume full proliferation but a subset of these cells were able to continue dividing, suggesting a partial irreversibly arrested cell population. Consistent with our results in Fig. 1A–B, no proliferation was observed when cells were switched back to full media after 14 days in CSS culture (Fig. 1D). Thus, collectively, our results verify that AD induces the major features of cellular senescence in LNCaP cells.

The ADIS phenotype we observe in LNCaP cells was preceded by progressively increasing total cellular ROS levels, observed as early as one day after switching cells to CSS media (Fig. 1E), as well as by the appearance of progressively greater numbers of co-localized gamma-H2AX and 53BP1 foci (Fig. 1F). These foci are indicative of a mounting DNA damage response (DDR), presumably in response to persistent unrepaired DNA damage [28], [38]. The ADIS-associated elevation in ROS levels and DNA damage foci are consistent with the known etiology of cellular senescence [36], further reinforcing that AD generates upstream stresses known to trigger cellular senescence.

### ADIS is Mediated by the p16^INK4a^ Pathway and Associated with Suppression of the p53 Pathway

**Figure 2:**
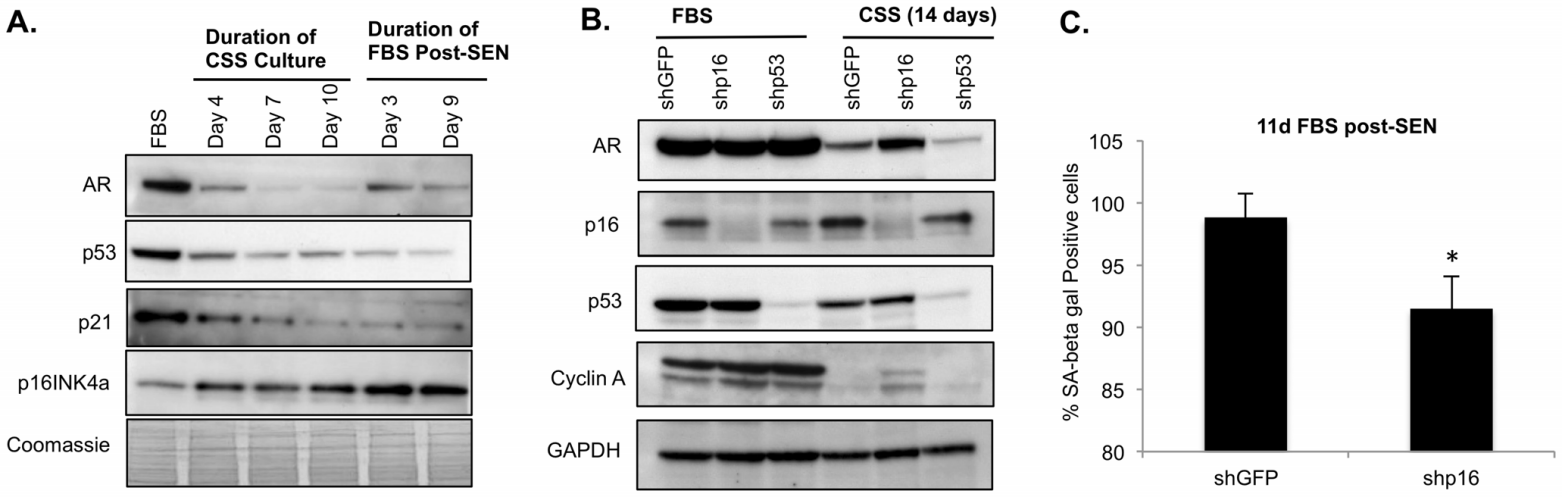
ADIS is mediated by the p16INK4a pathway. (**A**) Immunoblotting was carried out on approximately 35 µg of the indicated protein samples with antibodies against the denoted proteins. Coomassie blue staining was used to normalize for total protein loading. Note that AR expression decreases as expected upon CSS culture. The trend of p53, p21 and p16 protein expression is maintained following restoration of androgen-replete culture post-senescence (post-SEN) indicating the permanency of the changes. (**B**) Immunoblotting to show effects of shRNA-mediated knockdown of p16 on ADIS. Note elevated AR and cyclin A levels in shp16 cells under CSS culture relative to control or shp53 knockdown cells. (**C**) Effect of p16 suppression on SA-beta-gal staining. The indicated cells were cultured in CSS for 14 days and then switched to FBS- containing media for a further 9 days. Note the observed decrease in SA-beta-gal staining in shp16 cells. * p<0.05.

Two major tumor suppressor pathways, the p53/p21^Cip1/Waf1^ and p16^INK4a^, typically mediate senescence [39], [40]. Depending on the cell type or stressor, the senescent phenotype is associated with activation of one or both these pathways [39], [41]. However even if both pathways are activated, one may predominate to enforce the senescent phenotype [28], [40]. Accordingly we assayed LNCaP cells at increasing durations of CSS culture by probing total protein lysates from these samples for levels of the senescence-mediating proteins, p16^INK4a^ and p53/p21^Cip1/Waf1^. As expected, AR expression decreased with increasing duration of CSS culture (Fig. 2A). Although, *a priori*, the existence of a persistent DDR (Fig. 1F) led us to expect that the p53/p21 pathway would be involved, we found instead that ADIS was associated with increasing expression of p16^INK4a^ (Fig. 2A). Surprisingly levels of p53 and its effector cell cycle-regulatory protein, p21^Cip1/Waf1^, decreased with increasing duration of CSS culture (Fig. 2A). Furthermore, addition of 10 nM dihydrotestosterone (DHT) to CSS media was able to prevent both the observed decrease in AR and cyclin A levels and the ADIS-associated changes to p16^INK4a^ and p53 expression (Fig. S3A). This finding verifies these molecular changes occur specifically due to the absence of androgen in the culture media.

Significantly, p16^INK4a^ expression levels remained high and p53/p21 remained low in the bulk population even after androgen-replete culture was restored (Fig. 2A). This observation supports the permanence of the senescence-associated molecular circuitry activated by androgen-deprived culture despite the partial recovery of AR expression levels (Fig. 2A). Elevated p16^INK4a^ levels along with elevated SA-beta-gal staining, reduced proliferation and decreased AR and cyclin A expression were also observed following CSS culture of the androgen-responsive but p53-mutant [32] LAPC4 cell line (Fig. S4A-D). These findings underscore the functional importance of p16^INK4a^ in mediating the ADIS phenotype. To verify the physiologic importance of the above result, we examined human prostate sections consisting of normal, benign, or tumor-derived tissue (Fig. S5). Using Ki67 as a proliferation marker, we carried out immunohistological analyses of these sections for nuclear p16^INK4a^ and p53. In the majority of examined sections, particularly in normal or benign tissue, the extent of Ki67 corresponded inversely with nuclear p16^INK4a^ expression, with p53 levels either being low or absent (Fig. S5). Conversely, high Ki67 was detected in the absence of nuclear p16^INK4a^ staining and high p53 levels (Fig. S5), suggesting an uncontrolled proliferation stimulus [42] or missense p53 mutations typical of advanced disease [43], [44]. Thus our immunohistochemical studies confirmed p16^INK4a^ as the major regulator of cell proliferation in human prostatic tissue, supporting our observations in the LNCaP and LAPC4 cell culture models (Fig. 2A, Fig. S4C).

Although p16^INK4a^ levels were elevated with increased duration of CSS culture and also persisted despite restoration of androgen-replete conditions (Fig. 2A), we found that shRNA-mediated suppression of p16^INK4a^ (Fig. 2B) prior to androgen-deprived culture was not sufficient to fully prevent ADIS. We observed small increases in cell numbers for shp16^INK4a^ cells under CSS culture relative to control LNCaP shGFP cells; however, in the short term (less than 2 months) these could not be quantitatively reproduced (data not shown). Nevertheless, consistent with our observation, the shp16^INK4a^ LNCaP cells exhibited elevated levels of AR protein and the proliferation marker cyclin A following 14 days of CSS culture relative to the control cells (Fig. 2B). Suppression of p16^INK4a^ also reduced persistence of SA-beta-gal activity upon restoration of androgen-replete culture (Fig. 2C) and facilitated outgrowth of cell colonies after approximately two to four months in CSS culture (Fig. S3B; images from a representative 72-day experiment are shown). Unfortunately the cells derived from these colonies and those from similar experiments did not survive attempts toward expansion beyond one or two passages (data not shown). Thus, while the p16^INK4a^ pathway likely mediates the non-proliferative aspects of ADIS, it does not appear to be the predominant initiating mechanism. By contrast, as the p53/p21^Cip1/Waf1^ pathway was already downregulated under AD (Fig. 2A), suppression of p53 expression did not further affect proliferation markers such as cyclin A or AR levels (shp53 lane, Fig. 2B) nor did it affect SA-beta-gal activity (not shown).

### Emergence of ADIS-resistant Cell Variants with Androgen-refractory Traits are Observed Upon Repeated Exposure to Senescence-inducing AD

**Figure 3:**
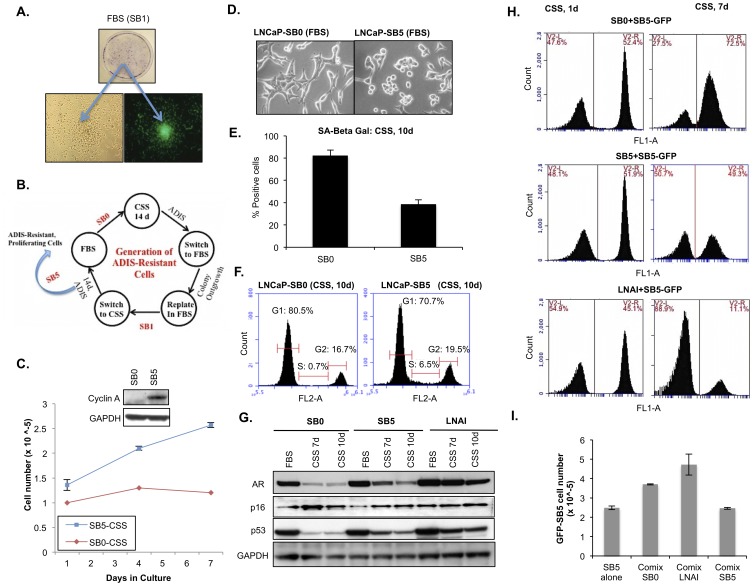
Repeated exposure of LNCaP cells to senescence-inducing stimuli via cyclic AD induces outgrowth of an androgen-refractory subpopulation. (**A**) Colony formation in GFP-labeled LNCaP cells and by crystal violet staining. Colonies indicates clones that exist in a transiently arrested state in the bulk population and resume division when androgen-replete conditions are restored after initial CSS culture. The indicated cells underwent one round of switch back to FBS media (SB1) following senescence in CSS culture. Following 21 days in FBS culture, clonal outgrowths were observed via crystal violet staining (∼0.1%). The insets show light microscopy and FITC images of a representative colony that has begun to proliferate in FBS media. (**B**) Schematic of the switch back (SB) method for generating androgen-refractory population of cells (designated LNCaP-SB5). (**C**) Proliferation curves for SB0 and SB5 cells in CSS. Cells were harvested on Day 7 and immunoblotted for cyclin A expression (inset) as a proliferation marker. (**D**) Morphology differences between LNCaP SB0 and SB5 cell populations. (**E**) Percentage SA-beta-gal positive cells: 87% LNCaP- SB0, 43% LNCaP-SB5. (**F**) PI cell cycle analysis (S phase: 0.7% LNCaP-SB0, 6.5% LNCaP-SB5). (**G**) Immunoblotting of 35 µg protein lysates from indicates samples against the denoted proteins. Note that AR expression remains elevated in SB5 versus SB0 cells. Androgen-refractory LNAI cells are shown as a positive control. GAPDH expression is used to normalize for protein loading. (**H**) Relative proliferation ability of SB5 in different co-cultures under AD. FL1-A indicates intensity of FITC signal and the right hand gate (V2-R) contains GFP-positive (SB5) cells whereas V2-L contains the co-mixed unlabeled cells. Representative flow profiles with the percentages (in red) of each co-mixed population are shown at the indicated time points. (**I**) Quantitation of GFP-positive (SB5) cell numbers under different co-culture conditions after 7 days of CSS culture are graphed. Quantitation was accomplished via the cell counting parameters from the CFlow software for counterpart cytometric profiles to those shown in (I).

The observed elevation in ROS and DSB foci that precedes ADIS (Figs. 1E, F) suggests that ADIS may be a form of stress-induced premature or accelerated cell senescence [45]–[47]. A common feature of this type of senescence is the eventual outgrowth of resistant or nonresponsive cells from the bulk senescent population, particularly in cancer cells. We found that although ADIS was maintained in the bulk population even after restoration of androgen-replete culture (Figs. 1A–C, 9d post-SEN; Fig. 2A), a small percent (∼0.1%) of cells were able to form colonies in androgen-replete media (denoted as the SB1 population for the first switchback (SB) to replete media; Figs. 3A, B) after approximately 3 to 4 weeks of culture.

The initial colonies that arose likely represent a reversibly arrested subset of cells, as they resumed proliferation once androgen-replete culture was restored. However the SB1 LNCaP cells could not proliferate under CSS culture (data not shown) and thus did not comprise an ADIS-resistant population. Nevertheless, we found that with each subsequent cycle of ADIS induction via CSS culture followed by restoration of androgen-replete media (Fig. 3B), these colonies appeared at earlier time points post-androgen restoration and at greater incidence. After the fifth androgen deprivation/restoration cycle (Fig. 3B), the outgrown cells (denoted SB5) were able to proliferate in CSS media and exhibited elevated cyclin A levels (Fig. 3C). This proliferative advantage of SB5 cells over parental (SB0) LNCaP was only observed under CSS culture, not under androgen-replete conditions (data not shown). The SB5 cells also exhibited markedly different morphology from the SB0 LNCaP cells (Fig. 3D), and more closely resembled fully androgen-refractory LNAI cells (Fig. S6A). The SB5 cells also showed reduced senescence markers relative to the parental LNCaP viz. ∼50% fewer SA-beta-gal positive cells following 10 days in CSS culture (Fig. 3E). Consistent with these observations, following 10 days of CSS culture, we observed a nearly 10-fold increase in S-phase cells, from 0.7% to 6.5% (Fig. 3F) and ∼50% lower p16^INK4a^ protein levels (Fig. 3G). As with the LNAI cells, the SB5 variants maintained higher levels of p53 expression under AD relative to SB0 cells (Fig. 3G). Significantly, unlike the parental SB0 LNCaP cells, the SB5 cells were able to partially maintain AR expression under CSS culture (Fig. 3G). It should be noted that the SB5 population was still clearly a mix of androgen-responsive and androgen-refractory cells judging by the differences in S-phase percentage between SB5 (6.5%) versus LNAI cells (17.8%) under CSS culture (Fig. 3F vs. Fig. S6B), and by the lower AR expression under CSS relative to LNAI (Fig. 3G). Nevertheless, overall, the LNCaP SB5 cells exhibited greater qualitative similarity in terms of morphology and molecular AD response to the established androgen-refractory LNCaP-variant LNAI than to the parental androgen-responsive LNCaP cells.

In order to determine whether the bulk senescent population was able to restrain or enhance the ADIS-resistant variants, we generated stable GFP-labeled LNCaP SB5 cells. Equal numbers of GFP-SB5 cells were co-mixed with unlabeled SB0, SB5 or LNAI cells, and after 7 days in CSS culture, the relative percentages and total numbers of GFP-positive cells (i.e. SB5-GFP) were determined (Figs. 3H, I). By Day 7 of CSS culture, the percentage of GFP-positive cells was approximately three times higher than the co-mixed unlabeled SB0 cells (72.5% SB5-GFP vs. 27.5% SB0, Fig. 3H, top panel), underscoring the relative proliferative advantage of the SB5 cells over the SB0 parental cells under AD. The number of unlabeled cells in the SB0+ SB5-GFP co-mix did not change substantially from Day 1 to Day 7 in CSS culture (Fig. S6E), confirming that AD inhibited proliferation of the LNCaP SB0 cells even in the co-mixed culture. By comparison, the percentages of co-mixed GFP-positive SB5 and unlabeled SB5 cells were essentially identical (50.7% vs. 49.3%, Fig. 3H, middle panel) indicating that the proliferative advantage of SB5-GFP cells in the SB0+SB5-GFP co-mix was not due to an artifactually enhanced growth rate resulting from the transduced GFP-expressing vector. As expected, the LNAI cells dominated the co-culture with SB5-GFP following 7 days in CSS media (88.9% vs. 11.1%, Fig. 3H, bottom panel), emphasizing that the SB5 cells represent a precursor androgen-insensitive variant relative to an established androgen-refractory cell line.

We also measured actual numbers of SB5-GFP cells at 7 days of CSS culture from the flow cytometric profiles of the various co-cultures to determine whether the co-mixed unlabeled cells affected their proliferation rate relative to equivalently seeded SB5-GFP cells cultured alone. We found that both the non-proliferating SB0 and the rapidly proliferating LNAI cells (Fig. S6E) increase the base proliferative rate of the SB5-GFP cells by a factor of 1.5 and 2-fold, respectively (Fig. 3I). As expected, there was no difference in proliferative rates between the SB5-GFP cells cultured alone and those co-cultured with unlabeled SB5 cells (Fig. 3I). The relatively similar degree of pro-proliferative effect produced on CSS-cultured SB5-GFP cells by the senescent SB0 cells and the rapidly proliferating LNAI cells suggests that the ADIS cells are capable of recapitulating a fully androgen-refractory cellular microenvironment. Thus, collectively, our results indicate that ADIS produces conditions that enhance the outgrowth of the ADIS-resistant androgen-refractory subpopulations.

We also obtained ADIS-resistant androgen-refractory outgrowths from LAPC4 cells within a single androgen depletion/repletion cycle (Fig. S4); although it should be noted that in contrast to the LNCaP SB0 cells, the parental LAPC4 SB0 cells significantly reduced their proliferation rate but did not completely enter a proliferative arrest under AD (Fig. S4B). This has been previously reported [48]. However, similar to LNCaP SB5 cells, the SB1 LAPC4 variants also showed markedly higher AR and cyclin A expression relative to the parental SB0 LAPC4 cells upon CSS culture (Fig. S4D). It should be noted that, unlike the LNCaP cells, LAPC4 possess mutant p53 [32] as evident from their elevated baseline p53 expression and the absence of AD-induced reduction in p53 levels (Fig. S4D). The lack of appropriate p53 function alone is not responsible for the relatively rapid emergence and/or expansion of an androgen-refractory LAPC4 population because we do not see a similarly accelerated emergence and expansion of ADIS-resistant subsets in shp53-transduced LNCaP cells (data not shown). However, the R175H p53 mutation in LAPC4 is a gain-of-function mutation [32] reported to promote cell proliferation and survival [49]–[52], thus potentially increasing the fraction of cells that resist ADIS.

Collectively, our results indicate that repeated application of ADIS-inducing stimuli leads to the selection and enrichment of an androgen-refractory cell subset within the bulk androgen-responsive parental population. The culture conditions utilized by us recapitulate intermittent AD therapy, a clinical regimen designed to improve patient quality of life but which has been reported to increase progression to castration-resistant disease in a subset of patients who underwent higher frequency of cyclic AD within the first 40 weeks [53].

### ADIS is Associated with a Pro-survival and Pro-inflammatory Phenotype

**Figure 4:**
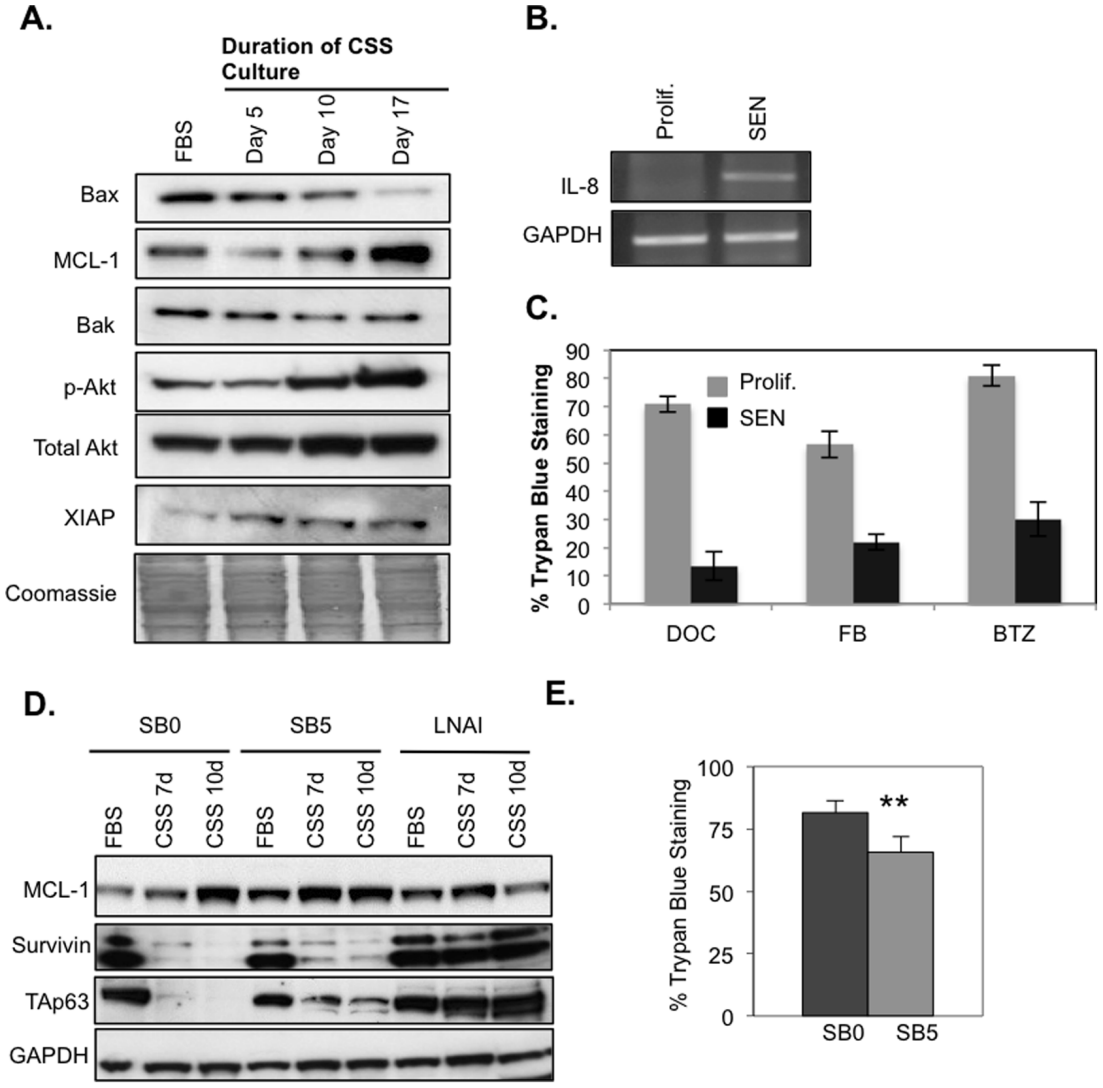
Senescent LNCaP cells display a pro-survival phenotype and upregulate IL-8 mRNA. (**A**) Western blot of survival markers in LNCaP cells growing in FBS and CSS day 5, 10 and 17. Cells were cultured under indicated conditions and 50 µg proteins immunoblotted against the indicated antibodies. Note that pro-apoptotic factor Bax is decreased and pro-survival factors MCL-1 and phospho-AKT are increased. Coomassie blue staining is used to normalize for loading. (**B**) RT-PCR showing IL-8 message levels in the indicated samples. ‘SEN’ samples were cultured in CSS media for 10 days to senescence. ‘Prolif’ indicates samples cultured in parallel in FBS media. (**C**) Trypan blue staining to indicate cell death in response to indicated treatments for 48 hours (n = 3): Docetaxel (1 nM, Doc); Flavopiridol (0.5 µM, FP); Bortezemib (1 µM, Btz). LNCaP cells were kept in CSS culture for 10 days prior to treatment. (**D**) Western blotting for pro-survival proteins from the indicated lysates. Note elevated Mcl-1, TAp63 and survivin levels in SB5 cells relative to SB0 cells. The TAp63 band corresponds to the 77 kDa alpha isoform; the other lower mobility isoforms are negligible or absent. (**E**) Trypan blue staining to indicate cell death. LNCaP SB0 and ADIS–resistant LNCaP SB5 cells in FBS media were treated with 1 nM Doc for 48 hours (n = 6), ** p<0.01.

Cell senescence is associated with reduced susceptibility to cell death in a number of different cell types [54]–[56]. Given that, post-AD, androgen-refractory cells arise from the initial growth-arrested population [15], [18], resistance to cell death is likely to encourage outgrowth and persistence of such androgen-insensitive cells. Accordingly we wanted to address two questions, namely, whether ADIS is also associated with elevated anti-apoptotic mechanisms and whether these mechanisms are retained or imprinted in the ADIS-resistant SB5 cells. We assayed for expression levels of the major cell death pathway proteins and found that as the LNCaP cell population progressed to ADIS under CSS culture, the p53-mediated pro-apoptotic protein Bax was downregulated whereas two anti-apoptotic proteins, Mcl-1 and XIAP (X-linked inhibitor of Apoptosis) were progressively elevated (Fig. 4A). The pro-apoptotic protein Bak, a target of Mcl-1, was also downregulated (Fig. 4A). We also found that Akt signaling, as determined by phospho-Akt (p-Akt) levels was enhanced with increasing duration of CSS culture (Fig. 4A). Cell senescence has also been associated with elevated pro-inflammatory cytokines [57]. We found that ADIS induced production of IL-8 (Fig. 4B), a pro-maligancy and proliferation-inducing cytokine in prostate cancer [58]–[60] and a key marker for the senescence-associated secretory phenotype [57], [61]. Consistent with our results in the non-senescent LNCaPs (Fig. 4B), it has been previously reported that IL-8 is negligibly present in these cells [60]. IL-8 has also been reported to inhibit chemotherapy-induced apoptosis in PC cells, including LNCaP [62].

To determine whether the above-mentioned ADIS-related changes were functionally associated with a pro-survival phenotype, we subjected LNCaP cells to CSS culture for over 10 days to establish senescence, and then compared induction of cell death by three functionally different, clinically relevant therapeutics, docetaxel (Doc), flavopiridol (FP) and bortezomib (BTZ) relative to androgen-replete cells. We found a significant reduction in drug-induced cell death for all three treatments in the former population relative to the latter (Fig. 4C). These results correlated with lower cleaved PARP (cl-PARP) levels in the respective post-SEN samples (Fig. S7A).

We next wanted to determine whether the LNCaP SB5 cells retained any of these characteristics conferred by the senescent state. Baseline expression levels of pro-survival markers Bcl-2, Mcl-1 and XIAP progressively increased in going from SB0 to SB5 LNCaP cells under androgen-replete culture (Fig. S6C). Of these, levels of Mcl-1 protein were maintained at higher levels in SB5 cells and in the established androgen refractory line LNAI relative to the SB0 LNCaP cells even under androgen-replete FBS culture (Fig. 4D). Bcl-2 and XIAP levels in SB5 cells behaved similarly to SB0 cells (not shown). However expression of the antiapoptotic protein survivin, clinically correlated with advanced and AD-resistant PC [63], [64], was higher under CSS culture in SB5 and in the LNAI cells versus the parental SB0 cells (Fig. 4D) and in the ADIS-resistant LAPC4 cells (Fig. S4D). Intriguingly, TAp63, the basal prostate progenitor cell marker [65] and inhibitor of premature senescence [66], [67], is expressed in SB5 LNCaP, SB1 LAPC4 and the fully androgen-refractory LNAI cells under CSS culture (Fig. 4D, Fig. S4D) whereas it is absent in the parental SB0 cultures that underwent ADIS (Fig. 4D, Fig. S4D). This observation suggests that TAp63 expression may serve as an early marker for androgen-refractory populations. By contrast, the alternate N-terminus deleted (deltaN) isoform of p63 is higher in SB0 LNCaP cells under CSS culture when compared to the SB5 or LNAI cells (Fig. S6D), suggesting it is subject to a different and possibly inverse regulatory mechanism relative to TAp63 in androgen-responsive LNCaP.

We further found a small but significant resistance to Doc-induced cell death in SB5 LNCaP cells under androgen-replete culture (Fig. 4E). No significant difference was observed between SB0 and SB5 cells for FP and BTZ treatment (data not shown). In regards to this finding, it should be noted that the proliferation, AR expression and cell cycle data all indicate that, under FBS culture, the SB5 population likely represents an admixture of fully androgen-refractory cells as well as parental-like androgen-responsive LNCaP cells. Thus the small survival advantage to Doc observed under replete culture (Fig. 4E) may only be representative of the small fraction of fully ADIS-resistant cells present within the SB5 bulk culture.

### AR Knockdown Induces Senescence in LNCaP SB0 Cells but not in the Androgen-refractory SB5 Variants

**Figure 5:**
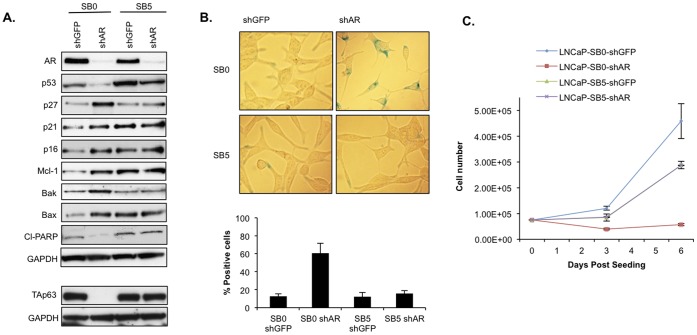
AR knockdown induces senescence in the LNCaP SB0 but not the LNCaP SB5 cells. LNCaP cells were transduced with the indicated shRNA constructs and kept continuously in 2 µg/ml puromycin selection, starting at 48 hours after transduction. (**A**) Western blotting for senescence-associated and pro-survival molecular markers. Six days following transduction with shGFP or shAR, SB0 and SB5 LNCaP cells were harvested and lysed. Prior to lysis, cells were kept continuously in 2 µg/ml puromycin selection, starting 48 hours transduction. Approximately 25 µg of protein was immunoblotted against the indicated antibodies. GAPDH expression was used to normalize for loading. The TAp63 signal is from the 77 kDa alpha isoform and the other two isoforms are absent. (**B**) SA-beta-gal assay. Cells were stained 7 days following transduction with shRNA constructs. Representative images are shown above the quantitation. (**C**) Proliferation rates. Cells were seeded in duplicate at a density of 7.5×10^4^ and counted at the indicated intervals. Note that the SB5 shGFP and shAR cells grow at near-identical rates, as expected.

Given the critical role of AR in prostatic cell proliferation and the observed decline in AR expression under CSS culture, we determined whether the loss of AR expression could recapitulate the salient features of ADIS. We observed that shRNA-mediated suppression of AR (Fig. 5A) in SB0 LNCaP cells resulted in a proliferative arrest and acquisition of senescence markers within a week of shAR transduction (Figs. 5A–C). However, AR knockdown in the SB5 LNCaP cells did not affect their proliferation or upregulate senescence markers (Figs. 5A–C). This finding underscores the androgen-refractory nature of the LNCaP SB5 variants as they are able to proliferate despite a significant reduction in AR expression. In the LNCaP SB0 cells, the shAR-induced senescent phenotype was accompanied by elevated p16^INK4a^ expression similar to the ADIS phenotype but also by elevated p21^cip1/waf1^ and p27^kip1^ levels, not observed in ADIS (Fig. 2A, Fig. S6D).

As seen with ADIS (Fig. 4A), shAR-induced senescence led to upregulated Mcl-1 expression (Fig. 5A) in the LNCaP SB0 cells. However, unlike ADIS, shAR-induced senescence was accompanied by upregulated Bak and Bax expression in the SB0 cells (Fig. 4A vs. Fig. 5A). However, the absence of upregulated cl-PARP levels (Fig. 5A) and lack of visual morphology indicating cell death (not shown) suggests that the elevated Mcl-1 expression is likely counterbalancing the pro-apoptotic stresses associated with AR knockdown. Consistent with our results in the LNCaP-SB0 cells that underwent ADIS (Fig. 4D), we find that shAR-induced senescence was also accompanied by loss of TAp63 expression. By contrast, as in the CSS-cultured SB5 cells (Fig. 4D), TAp63 expression is not lost in the shAR SB5 cells (Fig. 5A). AR knockdown led to reduced p53 expression in both SB0 and SB5 cells (Fig. 5A), suggesting that the observed decline in p53 expression under ADIS (Fig. 2A) likely results at least in part from AD-induced loss of AR expression.

### Sustained p53 Activation Prior to AD Induces Cell Death Instead of ADIS

**Figure 6:**
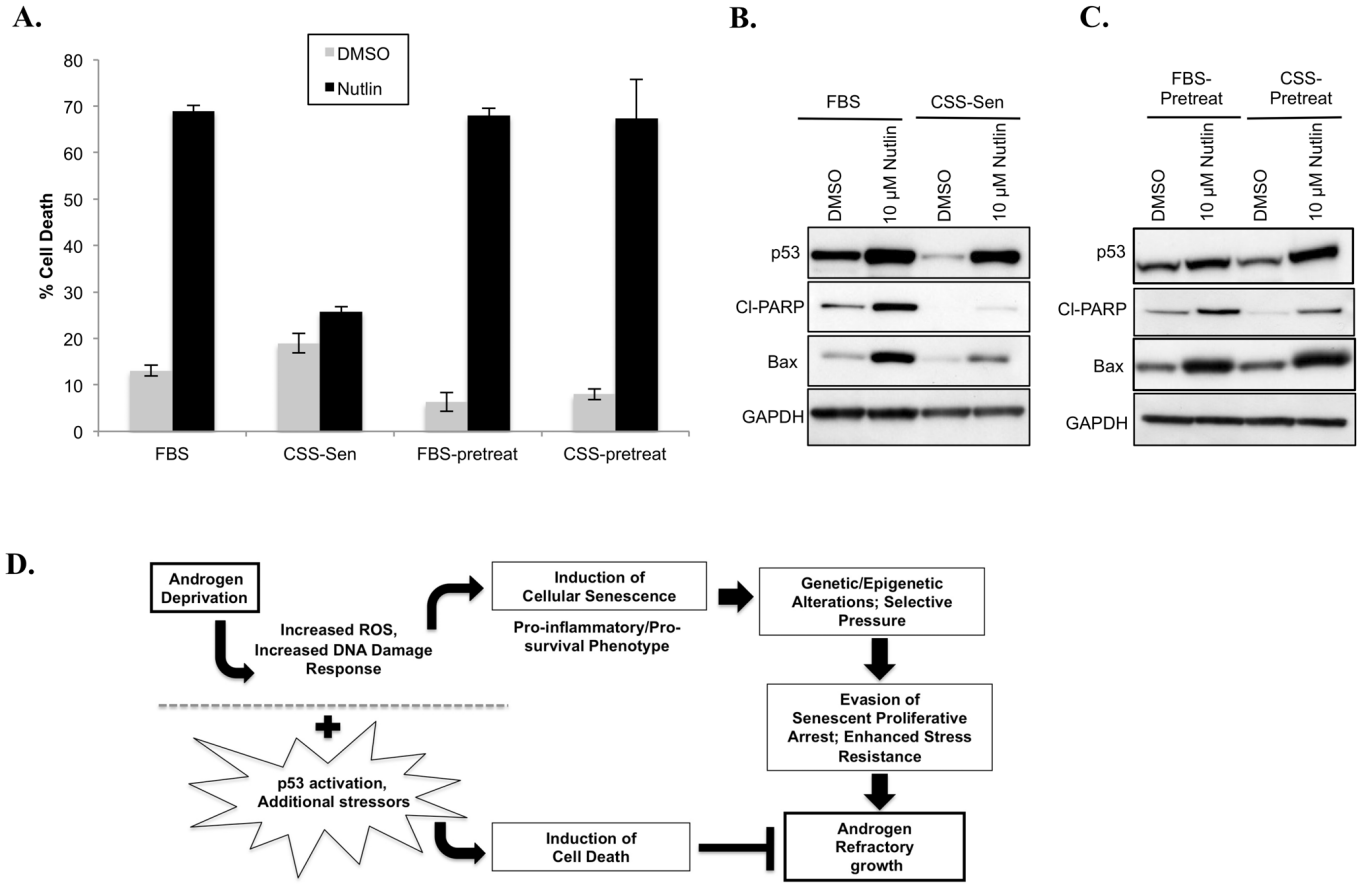
Activating p53 with Nutlin-3 prior to AD triggers cell death rather than senescence in LNCaP cells. (**A**) ADIS inhibits p53/Bax activation. LNCaP cells cultured in FBS or in CSS culture for 10 days till senescent (CSS-Sen) were subjected to 10 µM Nutlin-3 treatment for 72 hours. ‘Pre-treat’ indicates cells in FBS culture were pre-treated with a non-cytotoxic Nutlin-3 dose (2 µM, 48 hours) to activate p53 before being switched to fresh FBS or CSS as indicated and treated with 10 µM Nutlin for 72 hours. % Trypan blue staining indicates extent of cell death. (**B**), (**C**) Immunoblotting of counterpart samples from (A) against indicated proteins. Approximately 35 µg of samples were immunoblotted. Note increased p53, Bax and cl-PARP in Nutlin-3 treated compared to vehicle (DMSO) cells in LNCaP/FBS cells. Despite increased p53 expression in LNCaP/CSS-SEN cells, note the lower expression of cl-PARP and Bax under Nutlin-3 treatment when compared to the LNCaP/FBS cells. GAPDH expression is used to normalize for protein loading. (D) Schematic indicating stressors and tumor suppressor pathways evoked upon AD and the potential means of switching response from cell senescence to cell death.

Because the p53 pathway is involved in both cell senescence and cell death, one important implication of the AD-induced p53 decrease (Fig. 2A) is that while AD induces senescence, it may also promote resistance to cell death, potentially in the context of the AD-induced DDR (Fig. 1F). Thus, we wanted to determine whether the p53 pathway could be pharmacologically induced in androgen-deprived cells after they had achieved ADIS, to ascertain if such activation could trigger cell death. To do so, we utilized the Mdm2 inhibitor, Nutlin-3 [68]. While LNCaP cells in regular culture were very susceptible to Nutlin-3-induced cell death *via* the p53/Bax pathway (Figs. 6A, B; FBS), we found once the cells had attained ADIS following 10 days of CSS culture, they were unable to upregulate this pathway in response to Nutlin treatment (Figs. 6A, B; CSS). In this regard, they behaved similarly to LNCaP cells under FBS culture that had p53 expression suppressed via shRNA and thus showed minimal changes in p53, Bax or cleaved PARP expression upon Nutlin treatment (Fig. S7B, C). Significantly, if cells were pretreated with a non-lethal, non-senescence-inducing 2 µM Nutlin-3 dose for 48 hours prior to initiating AD and then moved to CSS culture, they were able to upregulate the pro-apoptotic protein, Bax, and were readily killed by this Nutlin dose during subsequent AD within 72 hours (Fig. 6A, C, pre-treated samples). This finding indicates that the inhibition of cell death is not a result of AD culture per se but rather due to the effect of ADIS and its associated p53/Bax downregulation. Thus our results support that sustained activation of p53 prior to and during AD is likely to improve the tumor-suppressive outcomes of AD by subverting cellular response from senescence to cell death (Fig. 6D).

## Discussion

In this study, we demonstrate that ADIS promotes pro-survival mechanisms in androgen-responsive PC cells and that repeated exposure to ADIS-inducing conditions facilitates outgrowth of ADIS-resistant subpopulations with androgen-refractory traits. Significantly, the ADIS-resistant LNCaP SB5 and LAPC4 SB1 subpopulations in our study exhibit molecular markers characteristic of clinical castration resistance such as elevated survivin [64] and AR levels [14] as well as increased levels of the prostatic basal stem cell marker, TAp63, upon AD (Figs. 3G, 4D, Fig. S4D). While the androgen-refractory and pro-survival molecular adaptations observed in the SB5 variants are pleiotropic traits, the observations that: 1) these variants are efficiently enriched from the bulk population through repeated selective pressure on cells able to evade ADIS (Fig. 3B), 2) have reduced ability to upregulate or sustain ADIS-specific markers (Fig. 3E–G), and 3) exhibit enhanced proliferation under AD in a senescent microenvironment (Fig. 3I) collectively suggest that senescence resistance is critically implicated in their emergence. This notion is further supported by the ability of these variants to maintain TAp63 expression under CSS culture in direct contrast to their parental lines (Fig. 4D, Fig. S4D), as TAp63 has been reported to inhibit premature senescence [66]. Accordingly, we postulate that the ADIS-resistant LNCaP SB5 and the LAPC4 SB1 cells represent the earliest androgen-refractory variants to arise from their respective parental androgen-responsive cells and thus comprise a valuable model system in which to investigate acute molecular adaptations underlying the etiology of androgen refractory PC growth.

Although our findings complement two recent studies that report AD-induced senescence in cell culture [23] and in animal models [24], our study further investigates the hitherto unexplored issue of whether senescence is a desirable tumor suppressor mechanism in androgen-responsive PC. The utility of a proliferation-arresting mechanism vis-à-vis pervasive cytotoxicity is limited in a relatively slow-growing tumor such as PC. In this context, the increasing knowledge that persistent senescent cells can also confer tumor-promoting properties [69], [70] needs to be considered when evaluating cellular outcomes of therapeutic regimens. Our results show that while ADIS does induce a permanent proliferative arrest in the bulk of androgen-responsive PC cells, it nevertheless promotes rapid expansion of androgen-refractory subpopulations that are able to evade ADIS while partially retaining ADIS-associated pro-survival traits (Fig. 4).

Judging by the relative differences in proliferation and molecular markers between the ADIS-resistant variants and the fully-androgen refractory LNAI cells, the ADIS-resistant cells are clearly part of a heterogenous bulk population consisting of senescent parental-like cells and the androgen-refractory senescence-resistant variants. Thus there may be a bystander effect exerted by the senescent cells in this population on the androgen-refractory SB5 variants. This type of bystander effect has been previously reported and can be either growth-suppressive [71], [72] or growth-promoting [73]–[75] to varying degrees. Our co-mix experiments in this study (Figs. 3H, I) suggest that the bystander effect of senescent prostate cancer cells on the proliferation of the androgen-refractory variants is enhancing rather than repressive. In fact, the senescent SB0 cells produce a proliferation-enhancing microenvironment effect similar to that of the fully androgen-refractory LNAI cells (Fig. 3I). Senescent cells have been reported to behave like reactive tumor stroma in their ability to confer pro-malignant effects on neighboring cells [76]–[78]. Indeed, a number of senescence-associated secreted factors are also found to be upregulated in prostate cancer-derived stromal cells [79] and actively influence disease progression (reviewed in [80]). Among these, IL-8 is prominently upregulated and has been reported to promote both prostate cancer cell proliferation and acquisition of androgen-refractory behavior [58], [59]. Although we have not comprehensively analyzed all senescence-associated secreted factors in our system, IL-8 stands out as being the most strikingly upregulated factor (Fig. 4B) in senescent but not in proliferating LNCaP cells. Thus our results herein suggest that a senescent milieu can not only exert selective pressures that promote outgrowth of androgen-refractory variants but can also support or promote the malignancy of such a population via senescence-associated paracrine factors.

The observation that TAp63 is present under CSS culture and AR knockdown in the ADIS-resistant variants (Fig. 4D, Fig. S4D, Fig. 5A) and in the fully androgen refractory LNAI cells (Fig. 4D) but not in the parental androgen-responsive lines (Fig. 4D, Fig. S4D) is intriguing. Another study has shown that elevated p63 expression appears to be a marker for a progenitor-like variant subpopulation isolated from the parental culture [81]. TAp63 marks basal prostatic cells, a progenitor population known to survive androgen ablation [82] and is involved in the maintenance of adult stem cells [66]. The differences in morphology, molecular response and TAp63 expression under AD or AR suppression in the SB0 vs. SB5 cells suggest that there may be a process of de-differentiation associated with androgen-refractory behavior. Alternatively TAp63 may be conferring a proliferative and/or survival advantage to cells that have lost AR signaling. Interestingly the alternate p63 isoform, deltaNp63, shows an inverse expression profile to TAp63 in SB0 LNCaP cells that undergo ADIS (Fig. 4D vs. Fig. S6D), consistent with the idea that TAp63 promotes cell plasticity whereas deltaNp63 enforces differentiation [83]. Most significantly, TAp63 expression has been associated with resistance to premature senescence [67], suggesting that maintenance of its expression under AD may be important for conferring ADIS resistance. Although further studies will be required to validate the role of TAp63 in the etiology of androgen resistance, our results support TAp63 as a potential early molecular marker for ADIS resistance and progression to androgen refractoriness.

Ultimately the incidence of androgen-refractory outgrowths would likely be greatly induced if the initial AD outcome could be subverted to pervasive and acute cell death instead of a proliferative arrest. It should be noted that the inherent cellular stresses induced by AD, namely the elevated ROS production and DNA damage response, (Fig. 1E, F) are capable of inducing cell death. The fact that senescence is instead induced is likely dependent on the downstream stress pathways that are activated in response to AD [84], [85]. In this context, the ADIS-associated upregulation of pro-survival factors (Fig. 4A), downregulation of p53 (Fig. 2A) and the inability to induce downstream p53 effectors, such as Bax (Fig. 6B) serve to abrogate a cytotoxic response to AD and potentially promote persistence of cells that eventually resist ADIS and comprise androgen-refractory outgrowths. In our study, as seen in both the senescing SB0 shAR LNCaP cells and the non-senescent shAR SB5 cells (Fig. 5A), the observed p53 decrease in ADIS appears to arise at least partially from loss of AR expression, although this decrease is more pronounced in the SB0 than the SB5 cells, suggesting the senescent phenotype may be enforcing or maintaining the reduction in p53 levels. However, comparing Figs. 2A and 4A with Fig. 5A, it is clear that not all the molecular circuitry of ADIS can be explained simply via AD-associated reduction in AR expression. Furthermore, the differences in molecular profile between the shAR SB0 and shAR SB5 cells (Fig. 5A) support that several key changes such as elevated Mcl-1 expression appear to be senescence- rather than AR-dependent. Indeed, AR expression has a complex relationship with senescence induction in prostate cancer cells, with a recent report showing that constitutive AR overexpression can also induce senescence, albeit via a p21-dependent rather than p16-dependent mechanism [86].

Nevertheless, the shAR-induced decrease in p53 is provocative. Several studies suggest that loss of p53 function may play a role in the acquisition of an androgen-refractory phenotype. For instance, it has been reported that reduction of wildtype p53 function confers androgen-refractory growth [87]. It is also known that p53 expression dampens the senescence-associated secretory response [57], suggesting that AD-induced p53 downregulation may be responsible for elevating levels of inflammatory cytokines such as IL-8 that, in turn, promote prostate cancer progression [58], [59]. It has been previously reported that concomitant upregulation of the p53 pathway in conjunction with AD is likely to yield better treatment outcomes than AD alone [88], [89]. While supporting the utility of p53 activation in conjunction with AD, our results additionally indicate that if such combinatorial treatment is not carried out in a timely manner, non-proliferating AD-treated cells lose or inhibit the ability to respond to p53-mediated cytotoxicity (Fig. 6A–C). Accordingly our results imply that combinatorial therapy using p53 activators prior to or concurrently with AD can enhance Bax-induced cell death in androgen-deprived PC cells, thus limiting the establishment of a population that facilitates progression to androgen-refractory tumors.

Collectively, our findings herein indicate that cell senescence as an AD response in PC cells is suboptimal and has the adventitious effect of promoting molecular changes that potentiate outgrowth of androgen-refractory subpopulations. To the best of our knowledge, this aspect of ADIS has not been previously reported, and provides a rationale for investigating senescence as a tumor-promoting mechanism in prostate cancer, particularly when induced by intermittent AD-utilizing therapeutic regimens. As senescence markers such as SA-beta-gal, reduced Ki67 or p16INK4a expression can be readily monitored in tissue samples, we suggest that advancement to androgen-refractory cancer may be clinically assessed by reduction or loss of these markers in AD-treated patient tumors.

## Supporting Information

Figure S1 **Bicalutamide treatment induces senescence. (A)** To establish proliferation curves, 1×105 LNCaP cells were plated on Day 0 and transferred on Day 1 to FBS or CSS-containing media or in FBS-containing media with 50 µM bicalutamide (Casodex). Per day, each sample was counted in triplicate for the total number of cells. Note that bicalutamide induces a proliferative arrest similar to CSS culture. (B) SA-beta-gal staining and quantitation was carried out as described in Figure 1. (C) Propidium iodide cell cycle analysis on LNCaP cells treated with bicalutamide for 10 days.
(PDF)

Figure S2 **The AD-induced proliferation arrest is irreversible in the bulk population once established.** LNCaP cells were cultured in either non-serum-containing media to induce quiescence (a transient proliferative arrest) or in CSS-containing media to induce ADIS. Cells were harvested at the indicated time points of treatment, and after 4 days following restoration of full culture medium in both sets of samples (indicated as SB for switch back to full serum culture). Approximately 35 µg of protein lysate was immunoblotted and probed for cyclin A levels as a molecular marker for proliferation. Note that cyclin A levels increase in the quiescent samples exposed to replete culture (Q SB) but not in the ADIS samples (CSS SB).
(PDF)

Figure S3 **Addition of dihydrotestosterone (DHT) to CSS media prevents ADIS-induced molecular markers. (A)** In order to determine whether the senescent-associated molecular circuitry is dependent on androgen deprivation, LNCaP cells were subjected to either culture in CSS media with DMSO or 10 nM dihydrotestosterone for the indicated durations. Cells were harvested and lysed for total protein and 35 µg protein was immunoblotted with antibodies against the indicated proteins. Note that addition of DHT prevents the AD-induced decrease in p53 and cyclin A and also prevents upregulation of p16. **(B)** Representative images of LNCaP samples under indicated culture conditions. Cells were plated in equivalent numbers (4×10^5^) in T75 culture flasks (VWR) and then switched to CSS culture after 24 hours. Media was changed every 3 days for the duration of cultures. Representative images are shown. Note the increased cell density indicating proliferation in the shp16 culture relative to the other samples.
(PDF)

Figure S4 **ADIS is observed in the androgen-responsive LAPC4 cell line.** LAPC4 cells were subjected to CSS culture as indicated. Following ADIS, cells were replaced in FBS media culture till proliferating outgrowths were observed, indicating transiently arrested cells denoted as SB1. **(A)** SA-beta-gal staining to indicate senescence. Note the lack of staining in SB1 cells under CSS culture. Representative images are shown from experiments run in duplicate. **(B)** Proliferation curves for the indicated samples. Note that the LAPC4 parental cells are not fully androgen refractory as seen from their low proliferative rate in CSS culture. **(C)** Immunoblotting the indicated samples indicates that SB1 cells have a higher baseline expression of p16 but show no further increase upon CSS culture. By contrast, the parental (SB0) cells show an increase in p16 expression consistent with establishment of senescence. **(D)** Comparison of key molecular markers differences in SB0 vs. SB1 LAPC4 cells under the indicated culture conditions. Approximately 35 µg protein was immunoblotted. Note the declining AR and cyclin A levels in parental LAPC4 cells and the constant expression of these markers in the ADIS-resistant SB1 LAPC4 cells. Note also the elevated TAp63 levels under CSS culture in SB1 cells.
(PDF)

Figure S5 Quantitation of Ki67, p16 and p53 staining in human-derived normal and prostate cancer tissue samples**. (A)** Representative histological images from stained tumor specimens. Tumor samples were obtained from the University of Miami Department of Pathology. All research involving human subjects has been approved by the University of Miami Institutional Review Board. The IRB approved waiver of consent for this protocol. Paraffin-embedded tissue blocks were provided, comprising 10 distinct samples. For histology, four sections were cut per block and mounted using Leica 2135 microtomes. Sections were stained with hematoxylin and eosin Y and with Ki67 (Dako, MiB-1), p53 (Dako, DO-7) or p16 (BD Pharmingen, 6175-405). Slides were processed using Dako Autostainer Plus. Slides were photographed at 40X using an Olympus DP71 camera mounted on a Windows computer. Formalin fixed paraffin-embedded samples from 10 patient cases were obtained from the Department of Pathology, with 10 slides from each comprising normal or benign tissue as well as tissue from tumors of Gleason grades 6, 7 or 8. Tissues were stained for Ki67, p53 and p16INK4a as described in Methods. **(B)** The intensity of staining in each section was scored as 0, 1, 2 or 3. Stacked plots are show for each scored slide. Note the inverse correlation between Ki67 and p16INK4a stain intensity. Also note, in general, p53 levels are elevated when Ki67 is elevated indicating a backup tumor suppressor response or dysregulated p53 response in advanced tumors.
(PDF)

Figure S6 Comparison of LNAi and LNCaP cells under AD culture. **(A)** Comparison of morphology between parental LNCaP SB0 and fully androgen-refractory LNAi cells. Note the smaller rounded shape of the LNAi cells, which resemble the appearance of LNCaP SB5 cells (see Fig. 3D). **(B)** Propidium iodide cell cycle analysis following 7 days in CSS culture for LNAi cells. Note the high percentage of cells in S-phase relative to the smaller percentage observed for SB5 cells. **(C)** Increasing baseline levels of pro-survival markers in going from parental to each successive SB outgrowth cells. Approximately 50 µg of protein was immunoblotted and probed against the indicated proteins. Note that the SB5 cells possess levels of these proteins comparable to the androgen-refractory LNAI line rather than the parental LNCaP cells from which they emerged. **(D)** Approximately 25 µg protein was immunoblotted and probed for deltaN p63 and p27 expression in LNCaP parental and variant cells as indicated. Note that the expression pattern for SB5 shows greater similarity to LNAI than to the SB0 LNCaPs. **(E)** Numbers of unlabeled cells respectively co-cultured with LNCaP SB5-GFP from the flow profiles quantitated in Fig. 3I. Cells were plated at 1×10^5^ on Day 0. Note relative lack of proliferation in SB0 cells.
(PDF)

Figure S7 **Effect of ADIS on chemoresponse and induction of the p53/Bax cell death pathway.** **(A)** Immunoblotting of samples from Fig. 4C against the indicated proteins. Note lack of cleaved PARP or p53 expression in the CSS-cultured samples. **(B)** Nutlin-3 treatment was carried out as described in Fig. 5A. Note that the shp53 samples show no upregulation of Bax or cl-PARP expression upon treatment. **(C)** Trypan blue staining from (B).
(PDF)
