# Supplementary figures and images for: Androgen Deprivation-Induced Senescence Promotes Outgrowth of Androgen-Refractory Prostate Cancer Cells
Source: PLoS One. 2013 Jun 28;8(6):e68003. doi: 10.1371/journal.pone.0068003 (PMC3695935; doi:10.1371/journal.pone.0068003)

**Figure S1**

**A.**

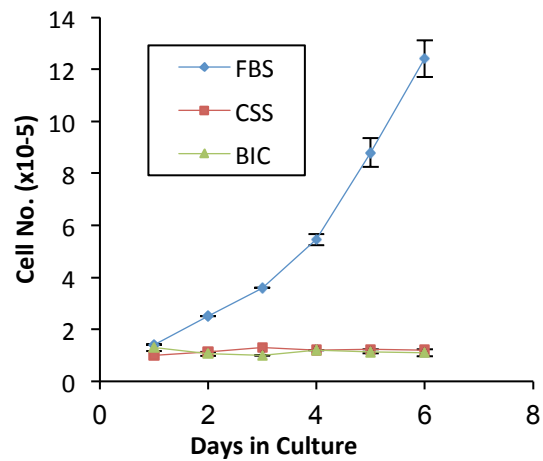

**B.**

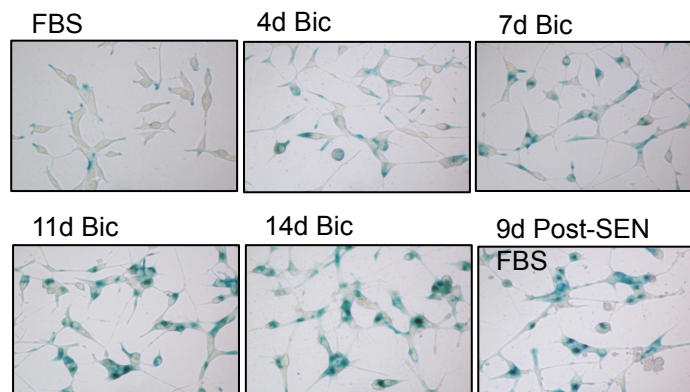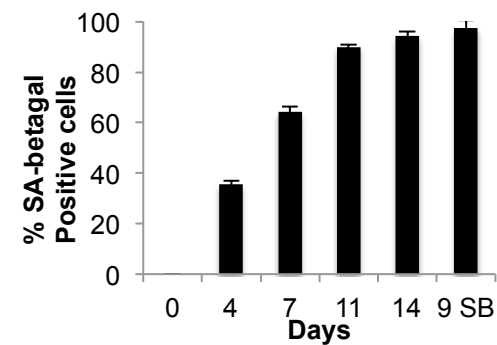

**C.**

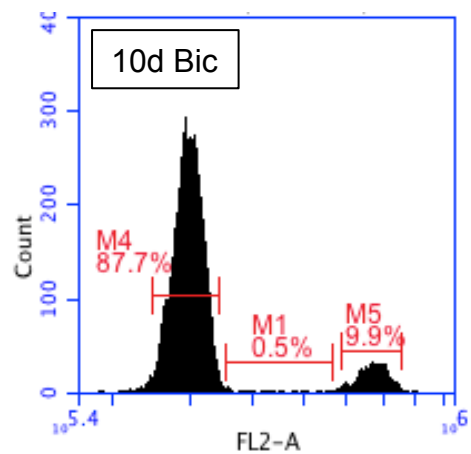

Supplement: Figure S1 — Bicalutamide treatment induces senescence. (A) To establish proliferation curves, 1×10^5^ LNCaP cells were plated on Day 0 and transferred on Day 1 to FBS or CSS-containing media or in FBS-containing media with 50 µM bicalutamide (Casodex). Per day, each sample was counted in triplicate for the total number of cells. Note that bicalutamide induces a proliferative arrest similar to CSS culture. (B) SA-beta-gal staining and quantitation was carried out as described in Figure 1. (C) Propidium iodide cell cycle analysis on LNCaP cells treated with bicalutamide for 10 days. (PDF) [file pone.0068003.s001.pdf]

### Figure S2

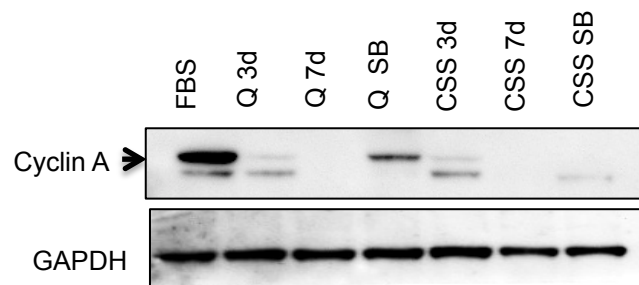

Supplement: Figure S2 — The AD-induced proliferation arrest is irreversible in the bulk population once established. LNCaP cells were cultured in either non-serum-containing media to induce quiescence (a transient proliferative arrest) or in CSS-containing media to induce ADIS. Cells were harvested at the indicated time points of treatment, and after 4 days following restoration of full culture medium in both sets of samples (indicated as SB for switch back to full serum culture). Approximately 35 µg of protein lysate was immunoblotted and probed for cyclin A levels as a molecular marker for proliferation. Note that cyclin A levels increase in the quiescent samples exposed to replete culture (Q SB) but not in the ADIS samples (CSS SB). (PDF) [file pone.0068003.s002.pdf]

A.

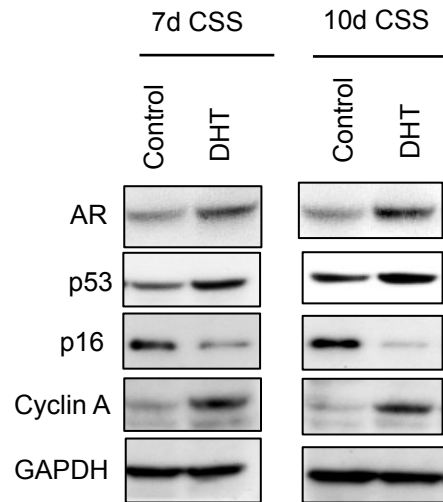

B.

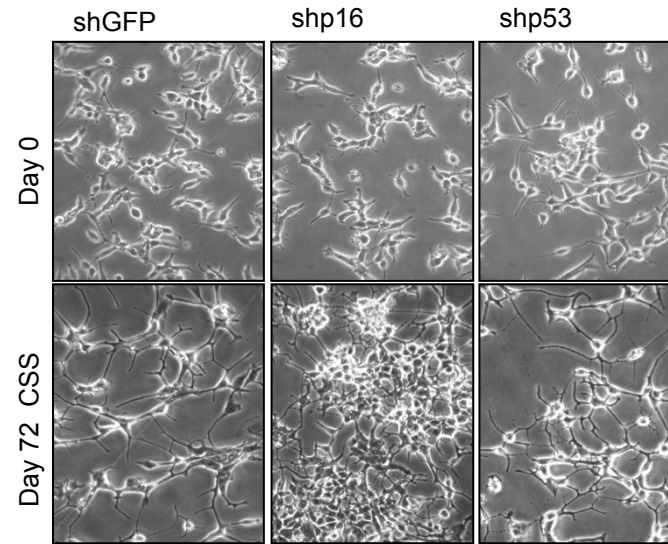

Supplement: Figure S3 — Addition of dihydrotestosterone (DHT) to CSS media prevents ADIS-induced molecular markers. (A) In order to determine whether the senescent-associated molecular circuitry is dependent on androgen deprivation, LNCaP cells were subjected to either culture in CSS media with DMSO or 10 nM dihydrotestosterone for the indicated durations. Cells were harvested and lysed for total protein and 35 µg protein was immunoblotted with antibodies against the indicated proteins. Note that addition of DHT prevents the AD-induced decrease in p53 and cyclin A and also prevents upregulation of p16. (B) Representative images of LNCaP samples under indicated culture conditions. Cells were plated in equivalent numbers (4×10^5^) in T75 culture flasks (VWR) and then switched to CSS culture after 24 hours. Media was changed every 3 days for the duration of cultures. Representative images are shown. Note the increased cell density indicating proliferation in the shp16 culture relative to the other samples. (PDF) [file pone.0068003.s003.pdf]

**Figure S4**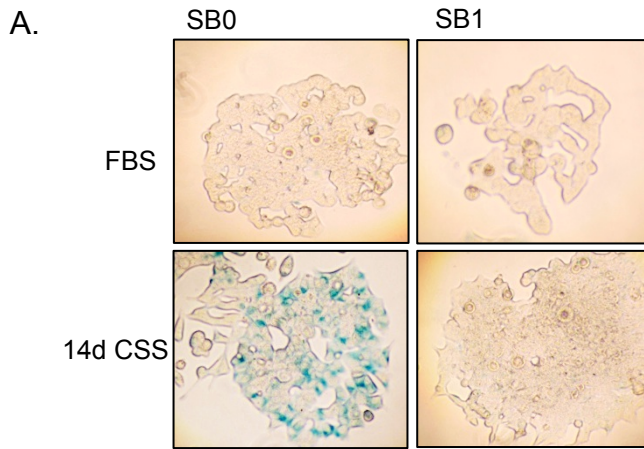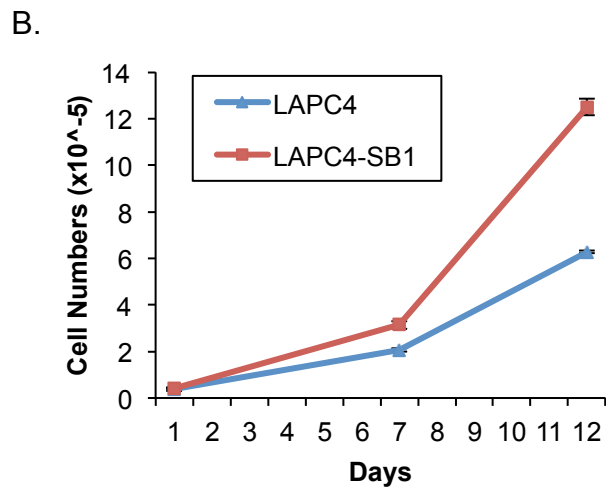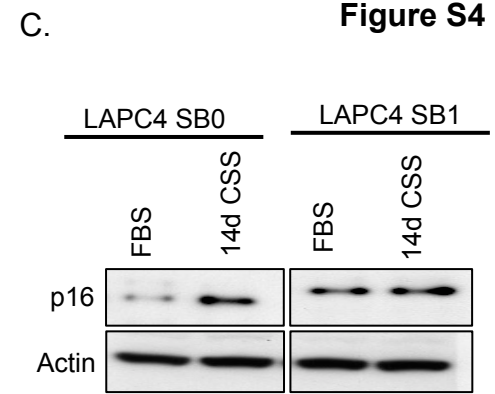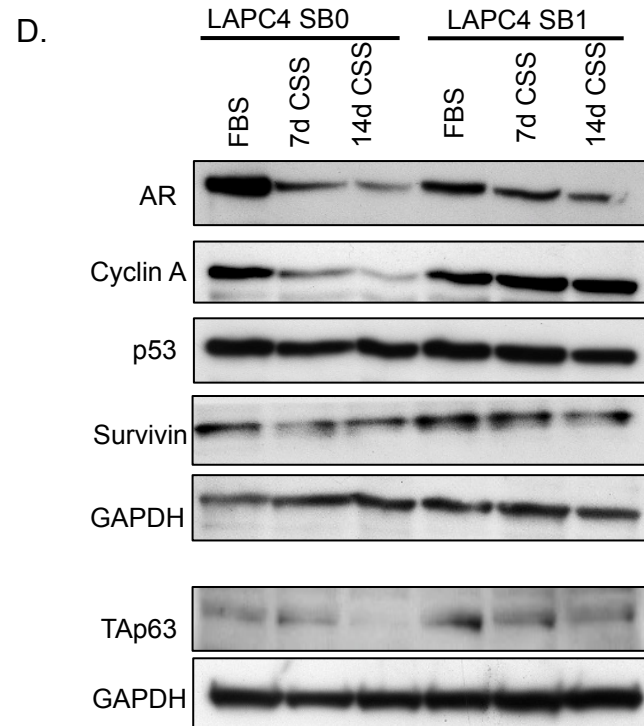

Supplement: Figure S4 — ADIS is observed in the androgen-responsive LAPC4 cell line. LAPC4 cells were subjected to CSS culture as indicated. Following ADIS, cells were replaced in FBS media culture till proliferating outgrowths were observed, indicating transiently arrested cells denoted as SB1. (A) SA-beta-gal staining to indicate senescence. Note the lack of staining in SB1 cells under CSS culture. Representative images are shown from experiments run in duplicate. (B) Proliferation curves for the indicated samples. Note that the LAPC4 parental cells are not fully androgen refractory as seen from their low proliferative rate in CSS culture. (C) Immunoblotting the indicated samples indicates that SB1 cells have a higher baseline expression of p16 but show no further increase upon CSS culture. By contrast, the parental (SB0) cells show an increase in p16 expression consistent with establishment of senescence. (D) Comparison of key molecular markers differences in SB0 vs. SB1 LAPC4 cells under the indicated culture conditions. Approximately 35 µg protein was immunoblotted. Note the declining AR and cyclin A levels in parental LAPC4 cells and the constant expression of these markers in the ADIS-resistant SB1 LAPC4 cells. Note also the elevated TAp63 levels under CSS culture in SB1 cells. (PDF) [file pone.0068003.s004.pdf]

A.

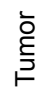

Tumor

Staining intensity

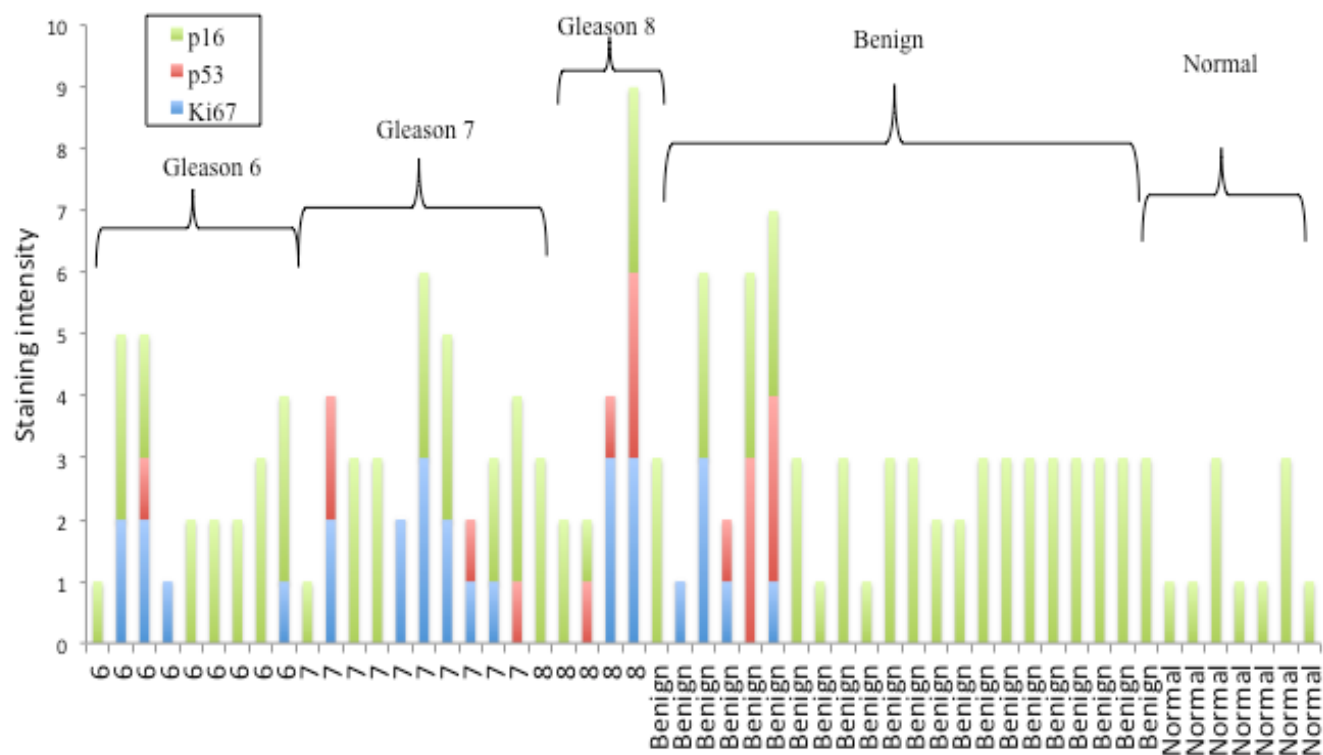

Supplement: Figure S5 — Quantitation of Ki67, p16 and p53 staining in human-derived normal and prostate cancer tissue samples. (A) Representative histological images from stained tumor specimens. Tumor samples were obtained from the University of Miami Department of Pathology. All research involving human subjects has been approved by the University of Miami Institutional Review Board. The IRB approved waiver of consent for this protocol. Paraffin-embedded tissue blocks were provided, comprising 10 distinct samples. For histology, four sections were cut per block and mounted using Leica 2135 microtomes. Sections were stained with hematoxylin and eosin Y and with Ki67 (Dako, MiB-1), p53 (Dako, DO-7) or p16 (BD Pharmingen, 6175-405). Slides were processed using Dako Autostainer Plus. Slides were photographed at 40X using an Olympus DP71 camera mounted on a Windows computer. Formalin fixed paraffin-embedded samples from 10 patient cases were obtained from the Department of Pathology, with 10 slides from each comprising normal or benign tissue as well as tissue from tumors of Gleason grades 6, 7 or 8. Tissues were stained for Ki67, p53 and p16INK4a as described in Methods. (B) The intensity of staining in each section was scored as 0, 1, 2 or 3. Stacked plots are show for each scored slide. Note the inverse correlation between Ki67 and p16INK4a stain intensity. Also note, in general, p53 levels are elevated when Ki67 is elevated indicating a backup tumor suppressor response or dysregulated p53 response in advanced tumors. (PDF) [file pone.0068003.s005.pdf]

A.

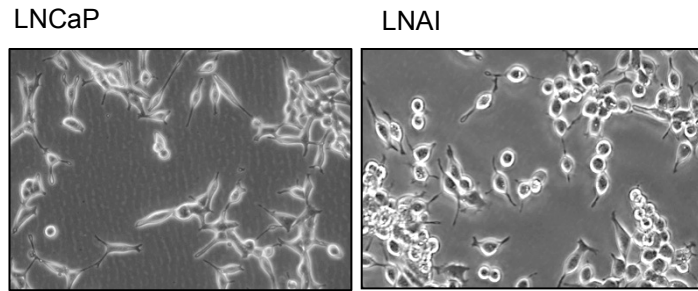

B.

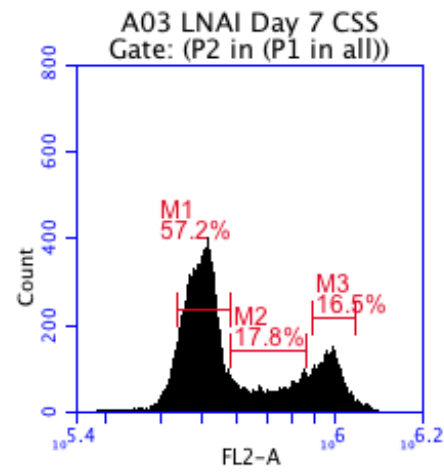

C.

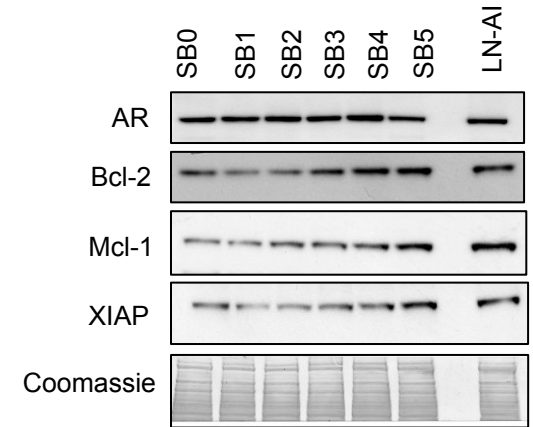

D.

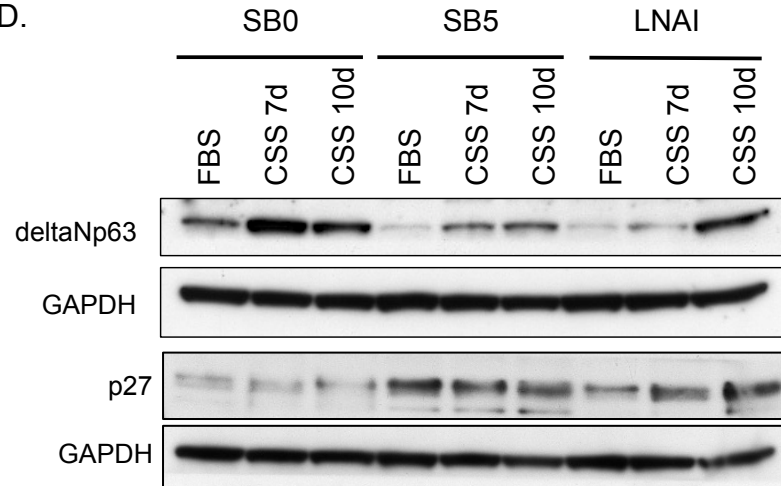

E.

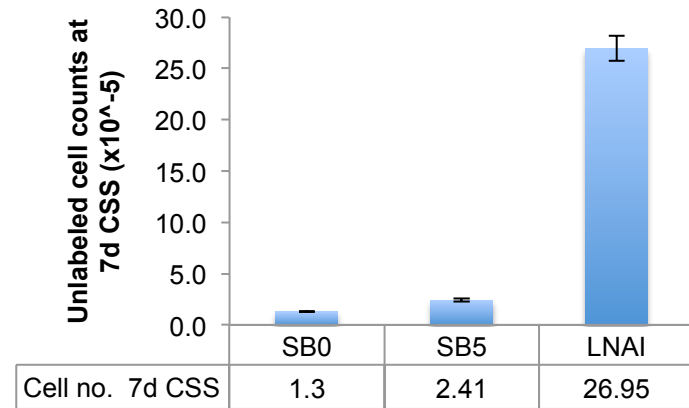

Supplement: Figure S6 — Comparison of LNAi and LNCaP cells under AD culture. (A) Comparison of morphology between parental LNCaP SB0 and fully androgen-refractory LNAi cells. Note the smaller rounded shape of the LNAi cells, which resemble the appearance of LNCaP SB5 cells (see Fig. 3D). (B) Propidium iodide cell cycle analysis following 7 days in CSS culture for LNAi cells. Note the high percentage of cells in S-phase relative to the smaller percentage observed for SB5 cells. (C) Increasing baseline levels of pro-survival markers in going from parental to each successive SB outgrowth cells. Approximately 50 µg of protein was immunoblotted and probed against the indicated proteins. Note that the SB5 cells possess levels of these proteins comparable to the androgen-refractory LNAI line rather than the parental LNCaP cells from which they emerged. (D) Approximately 25 µg protein was immunoblotted and probed for deltaN p63 and p27 expression in LNCaP parental and variant cells as indicated. Note that the expression pattern for SB5 shows greater similarity to LNAI than to the SB0 LNCaPs. (E) Numbers of unlabeled cells respectively co-cultured with LNCaP SB5-GFP from the flow profiles quantitated in Fig. 3I. Cells were plated at 1×10^5^ on Day 0. Note relative lack of proliferation in SB0 cells. (PDF) [file pone.0068003.s006.pdf]

A.

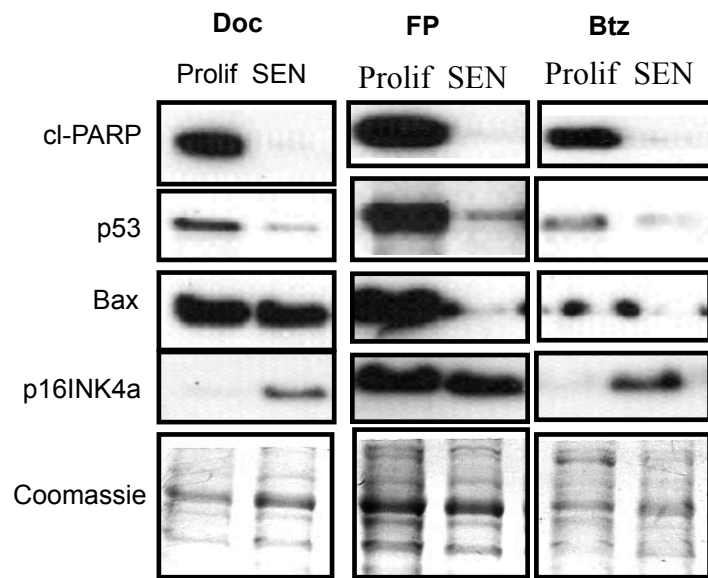

B.

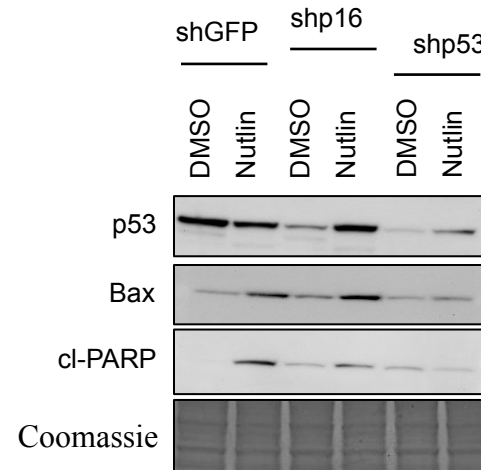

C.

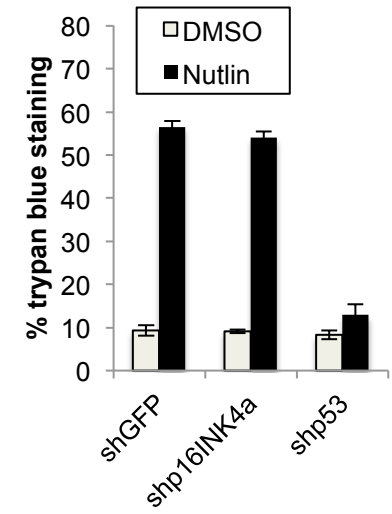

Supplement: Figure S7 — Effect of ADIS on chemoresponse and induction of the p53/Bax cell death pathway. (A) Immunoblotting of samples from Fig. 4C against the indicated proteins. Note lack of cleaved PARP or p53 expression in the CSS-cultured samples. (B) Nutlin-3 treatment was carried out as described in Fig. 5A. Note that the shp53 samples show no upregulation of Bax or cl-PARP expression upon treatment. (C) Trypan blue staining from (B). (PDF) [file pone.0068003.s007.pdf]
